# The regulatory role of endoplasmic reticulum chaperone proteins in neurodevelopment

**DOI:** 10.3389/fnins.2022.1032607

**Published:** 2022-11-15

**Authors:** Hongji Sun, Mengxue Wu, Minxin Wang, Xiaomin Zhang, Jia Zhu

**Affiliations:** ^1^School of Basic Medicine, Kunming Medical University, Kunming, Yunnan, China; ^2^Forensic and Pathology Laboratory, Department of Pathology, Institute of Forensic Science, Jiaxing University, Jiaxing, China

**Keywords:** neurodevelopment, endoplasmic reticulum, molecular chaperone, neuronal migration, neuronal morphogenesis, synaptic function

## Abstract

The endoplasmic reticulum (ER) is the largest tubular reticular organelle spanning the cell. As the main site of protein synthesis, Ca^2+^ homeostasis maintenance and lipid metabolism, the ER plays a variety of essential roles in eukaryotic cells, with ER molecular chaperones participate in all these processes. In recent years, it has been reported that the abnormal expression of ER chaperones often leads to a variety of neurodevelopmental disorders (NDDs), including abnormal neuronal migration, neuronal morphogenesis, and synaptic function. Neuronal development is a complex and precisely regulated process. Currently, the mechanism by which neural development is regulated at the ER level remains under investigation. Therefore, in this work, we reviewed the recent advances in the roles of ER chaperones in neural development and developmental disorders caused by the deficiency of these molecular chaperones.

## Introduction

Neurodevelopment is a highly complex and precisely regulated process (Bagni and Zukin, 2019). The establishment of the human embryonic central nervous system begins on the 22nd day after fertilization when the neural tube begins to form (Devakumar et al., 2018). As development progresses, the anterior portion of the neural tube expands to form “brain vesicles”, and the brain develops from five small vesicle walls filled with fluid (Jiang and Xu, 2019). The initial vesicle wall has only two layers: the compartment layer and the marginal layer (Bostan et al., 2018; Moon and Xiong, 2022). Neural precursor cells (NPCs) are located in the inner layer of vesicles, and cells in the ventricular layer proliferate. Some of the newly generated cells remain in the ventricular layer where they to continue to divide and differentiate. Finally, these cells proliferate and form different brain regional structures, such as the cerebral cortex, thalamus, and hippocampus (Trujillo et al., 2005; Turrero García and Harwell, 2017). Another group of daughter cells located furthest from the ventricle side begin to migrate outward. After these cells reach various regions of the brain and occupy specific locations, they lose their ability to divide, undergo cell differentiation, and finally form the brain (Crelin, 1974; Li and Pleasure, 2014; Belmonte-Mateos and Pujades, 2022).

At the cellular level, NPCs proliferate and differentiate into immature neurons (Riquelme et al., 2008). Then, immature neurons undergo a series of morphological changes, including neuronal axon and dendrite development (Omotade et al., 2018; Prigge and Kay, 2018), axonal myelination, and dendritic spine formation (Nimchinsky et al., 2002), and gradually become mature neurons. Neurons are interconnected by synapses formed by both axonal endings and dendritic spines, forming complex neural networks (Omotade et al., 2018; Kadoyama et al., 2019). Abnormalities in any of these processes may impair neural development, resulting in brain dysfunction and subsequent leading to brain diseases (Guerrini and Dobyns, 2014).

The endoplasmic reticulum (ER) is the largest tubular reticular organelle in eukaryotic cells (Rapoport, 2007; Pobre et al., 2019) and plays several essential roles, including Ca^2+^ storage and release, lipid synthesis, intracellular signaling, and protein synthesis (Phillips and Voeltz, 2016; Schwarz and Blower, 2016). ER molecular chaperones are a class of proteins ubiquitous in living organisms and are widely found in prokaryotes and eukaryotes (Doyle et al., 2013; Bose and Chakrabarti, 2017). These molecular chaperones are mainly responsible for assisting in correct protein folding, assembly, *trans*-location, degradation of misfolded proteins and inhibition of protein aggregation to maintain normal protein homeostasis (Carver et al., 2018; Freilich et al., 2018; Takeuchi, 2018; Ma et al., 2020).

Studies have found that abnormal expression of molecular chaperones often leads to abnormal neurodevelopment and even causes a variety of different neurodevelopmental diseases (Fatemi et al., 2005; Lammert et al., 2017). ER chaperones involved in neural development are mainly divided into the following categories: heat shock family proteins including heat shock protein 70 (HSP70) and heat shock protein 90 (HSP90) (Miller and Fort, 2018), glycan-binding lectin chaperones including calnexin (CNX) and calreticulin (CRT) (Ni and Lee, 2007), protein disulfide isomerase (PDI) (Turano et al., 2002), sigma-1 receptor (Sig-1R) (Su et al., 2010; Zhemkov et al., 2021; Dalwadi et al., 2022) and prefoldin (PFDN) (Siegert et al., 2000).

HSP70 and HSP90, members of the heat shock protein family, are highly conserved and ubiquitous molecular chaperones (Tsuboyama et al., 2018). The reversible interaction between HSP70 and peptides plays an important role in protein folding, transport, degradation of misfolded peptides and maintenance of cellular homeostasis (Fernández-Fernández and Valpuesta, 2018). HSP90 and its co-chaperones coordinate key physiological processes, such as cell survival, cell cycle control, hormone signaling, and apoptosis (Hoter et al., 2018). Binding immunoglobulin protein (Bip) and glucose-regulated protein 94 (GRP94) are the main HSP70 and HSP90 proteins in the ER. Bip cannot function alone, and the binding and separation between Bip and nascent proteins require the participation of other auxiliary proteins, such as the cochaperone SIL1, the main function of which is to serve as a nucleotide exchange factor (NEF) to assist Bip protein in binding nascent peptide chains and mediating their folding and assembly (Otero et al., 2010). However, GRP94 expression in the ER is mostly related to tumor cancer and other related diseases (Gewirth, 2016). CRT and CNX bind Ca^2+^ and act as molecular chaperones in the transition of proteins from the ER to the extracellular membrane and regulate Ca^2+^ balance in the ER (Tjoelker et al., 1994). In addition to assisting protein folding consistent with the Sig-1R and PFDN protein, PDI is also involved in the formation and isomerization of disulfide bonds to prevent intermolecular aggregation (Wilkinson and Gilbert, 2004).

In addition, in the ER, chaperones not only exert their respective functions individually but also interact with each other to maintain ER homeostasis and protein homeostasis. For example, glycan-binding lectin chaperones form the glycosylation-deglucosylation cycle. As a reticulin, CRT is involved in a quality control system for newly synthesized proteins and glycoproteins that relies on multiple additional chaperons, including CNX and protein disulfide isomerase family A member 3 (PDIA3) (Fucikova et al., 2021). This system is often referred to as the CNX/CRT cycle. The CNX/CRT cycle specifically recognizes glycoproteins linked by N2 glycosidic bonds and is an important monitoring mechanism for protein folding and assembly in eukaryotic cells as well as regulating Ca^2+^ homeostasis and Ca^2+^ signaling processes (Insert a) in the ER (Helenius and Aebi, 2001). As a highly conserved molecular chaperone, Bip assists in a wide range of folding processes through its two domains: the nucleotide binding domain (NBD) and the substrate binding domain (SBD) (Mayer and Gierasch, 2019). When adenosine diphosphate (ADP) binds the NBD, the SBD conformation changes, and the substrate binding affinity of Bip increases. The SBD lid closes on the bound substrate, limiting the conformational freedom of the substrate and protecting the bound substrate from premature folding or aggregation (Hendershot et al., 1996). The NEF of Bip can break the hydrogen bond contact and detach the bound ADP molecule (Tyson and Stirling, 2000) so that NBD can bind to the new adenosine triphosphate (ATP) molecule, and the substrate binding affinity is reduced. SBD docks on the NBD and opens the lid to release the bound polypeptide substrate (Insert b). At present, SIL1 and glucose-regulated protein (GRP170) are the two co-chaperones that can bind Bip to promote NEF activity (Yan et al., 2011). Bip is also a regulator of Ca^2+^ homeostasis in the ER, maintaining Ca^2+^ balance in the ER (Lièvremont et al., 1997). In addition, when unfolded/misfolded proteins in the ER exceed the capacity of the protein folding mechanism, Bip can initiate the unfolded protein response (UPR), assist in ER-associated degradation (ERAD), reduce the unfolded/misfolded protein load, and induce autophagy (Wang J. et al., 2017).

Recent studies have shown that many ER chaperone proteins participate in neural development. Therefore, in this work, we review the regulatory roles of ER chaperones in various stages of central nervous system development as well as recent research progress on related neurodevelopmental diseases.

## The role of binding immunoglobulin protein in neurodevelopment

Heat shock proteins are a large family of evolutionarily conserved molecular chaperones that play key roles in cell survival and development, and their expression is induced by different factors, including heat shock, nutrient deficiency, hypoxia, and intoxication (Tsuboyama et al., 2018). HSP70 family plays an essential role in protein folding, transport, degradation of misfolded polypeptides, and maintenance of cellular homeostasis (Fernández-Fernández and Valpuesta, 2018). Among them, Bip [also known as glucose-regulated protein 78 (GRP78) or heat shock protein A5 (HSPA5)] with a molecular weight of 78 kDa, mainly exists in the ER and is responsible for binding the nascent peptide chain and mediating its folding and assembly (Wang et al., 2009). Bip cannot perform its functions alone, but other auxiliary proteins are required for its binding and separation from nascent proteins (Otero et al., 2010). The effects of Bip on neurodevelopmental processes are mainly reflected in embryonic development, cortical neuron migration, and coordination of developmental processes in conjunction with some ER cochaperone proteins.

### Binding immunoglobulin protein regulates the migration and localization of developing cortical neurons

Mutant mice with Bip deficiency die during the embryonic period (Luo et al., 2006; Jin et al., 2017), and the main cause of death is abnormal expression of the pulmonary surfactant protein surfactant protein-A (SP-A) and prominently surfactant protein-C (ProSP-C), which subsequently triggers respiratory insufficiency in neonatal or newborn mice and leads to death. In addition to respiratory failure, Bip mutant mice exhibit abnormal neuronal migration and dysplasia. Normal cortical neurogenesis in the ventricular zone (VZ), and newborn neurons migrate to different layers of the cerebral cortex through radial migration (Nadarajah and Parnavelas, 2002; Marín et al., 2010). Neurons generated earlier migrate to the deep cortex, whereas neurons generated later migration to shallow layers (Ghashghaei et al., 2007; Kwan et al., 2012). The subplate (SP), cortical plate (CP), and marginal zone (MZ) are formed in an inside-out migration pattern during development (Caviness, 1982). However, newborn neurons in Bip mutant mice are the first to migrate to the MZ and remain there, whereas later-born neurons fail to migrate to the upper layers. The mutant brain exhibits an outside-in pattern during neocortical layer formation. This phenotype is observed because loss of Bip affects the expression of reelin, a protein that plays an essential role in neuronal migration. In wild-type mice, reelin-secreting Cajal-Retzius (CR) cells are located in the superficial layer of the cortex. In contrast, CR cells in mutant Bip mice are scattered around the upper layer of the neocortical primordium, and CR cells in Bip mutants do not secrete reelin. Therefore, Bip mutant mice exhibit a phenotype similar to that of reelin knockout mice (Mimura et al., 2008).

### Binding immunoglobulin protein regulates motor neuron development

Heterozygous mutant Bip mice exhibit no significant difference in lifespan compared with wild-type mice, but mutant Bip mice develop renal tubular-interstitial lesions as they age (Kimura et al., 2008). Some mutant Bip mice develop paralysis and tremors after 12 months. These mice exhibit motor nerve injury symptoms, such as loss of righting reflex, and suffer from paralysis (Jin et al., 2014). In addition, studies have shown that Bip deletion is associated with the neurodegenerative disease amyotrophic lateral sclerosis (ALS). ALS is a fatal neurodegenerative disease characterized by progressive degeneration of upper and lower motor neurons (Beghi et al., 2011; Filareti et al., 2017). In recent years, impaired protein homeostasis has been found to be a key factor in the pathogenesis of ALS (Zhao et al., 2008; Ruegsegger and Saxena, 2016), and Bip plays an important role in this process. Studies have found that Bip-deficient mice show more severe neurological decline and spinal motor neuron loss symptoms than normal mice. Gómez-Almería et al. created mutant superoxide dismutase 1 (mSOD1) and Bip double mutant (mSOD1/Bip+/-) mice to study the role of Bip in a mouse model of the neurodegenerative disease ALS (Gómez-Almería et al., 2021). They found that knockout of Bip show more intense neurological decline than mSOD1 single knockout mice. Then, to determine the relationship between the Bip protein and the pathogenesis of ALS, they also discussed the potential relationship between the Bip protein and the type-1 cannabinoid (CB1) receptor that mediates neuroprotection. CB1 receptor is mainly located in neurons of the central nervous system (Chiarlone et al., 2014). Numerous studies have reported that CB1 receptor-mediated neuroprotection is related to ALS (Abood et al., 2001; Rossi et al., 2010). They found that in mSOD1 mice with partial Bip gene deletion, the level of the CB1 receptor was significantly decreased, which indirectly indicated that Bip may play a neuroprotective role in ALS (Gómez-Almería et al., 2021). Similarly, Apolloni et al. found that increasing Bip and Hsp70 protein levels in the spinal cord and cortex of ALS model mice could rescue the loss of motor neuron dendritic spines (Apolloni et al., 2019). These studies suggest that Bip plays vital roles in motor neuron development.

### Binding immunoglobulin protein regulates the plasticity of the central nervous system

Studies have shown that the interaction between Bip and N-methyl-D-aspartate (NMDA) receptors may affect synaptic transmission. NMDA receptors play essential roles in brain development, plasticity, and pathology. Functional NMDA receptors are tetramers mainly composed of two GluN1 subunits and two GluN2 regulatory subunits (Cull-Candy et al., 2001; Yashiro and Philpot, 2008). The number and composition of GluN2A and GluN2B containing NMDA receptors at synapses are dynamically regulated during development and neuronal activation, which is thought to control synaptic plasticity (Nase et al., 1999; Lopez de Armentia and Sah, 2003; Liu et al., 2004). Using immunoprecipitation, the authors examined the distribution of Bip in neurons and found that Bip is strongly expressed in both cortical and hippocampal neuronal cell bodies and neurites. Furthermore, Bip co-localizes with GluN2A in cultured cortical neuron dendrites, and preventing the binding of Bip to GluN2A reduces synaptic transmission and impairs memory formation (Zhang et al., 2015).

### Binding immunoglobulin protein and SIL1 jointly regulate neural development

SIL1 is a 54-kDa ER protein composed of 461 amino acids and is a NEF for Bip (Wang J. et al., 2017). As an auxiliary protein of Bip, the synergistic effect of SIL1 and Bip has consistently been a research hotspot. The central nervous system of mice with SIL1 deficiency exhibits developmental defects, including abnormal neuronal morphological development, cortical neuron migration, and localization (Inaguma et al., 2014).

#### SIL1-binding immunoglobulin protein coordinately regulates neuronal migration

SIL1 plays a role in neuronal migration in the neocortex. Inaguma et al. constructed a SIL1 knockout plasmid, transfected it into the neural progenitor cells of the VZ of the embryonic mouse brain through *in utero* electroporation (Tabata and Nakajima, 2001; Nishimura et al., 2012) and observed the localization of the transfected cells at the post-natal day 0 (P0). In the control group, neurons normally migrated to the superficial layers (layers II-IV) of the CP, whereas the SIL1-deficient neurons remained in the middle or lower layers of the CP. Then, the authors performed immunostaining to assess the differentiation status of the abnormally localized cells and found that the abnormally localized cells remained in the immature neuronal state (NPCs or basal progenitor cells) (Inaguma et al., 2014).

Considering that SIL1 acts as a chaperone for Bip and regulates its function, Bip may also be involved in neuronal migration. Therefore, Inaguma et al. also examined the role of Bip in neuronal migration during cortical formation and compared it with SIL1. They found that a large fraction of Bip-deficient (through RNA interference) neural progenitor cells, which remain in the lower part of CP, intermediate zone (IZ), and subventricular zone (SVZ)/VZ at P0, exhibit a phenotype similar to that of SIL1-deficient cells. Bip-deficient neurons showed delayed migration at embryonic day 17 (E17) but localized in normal positions at post-natal day 7 (P7). However, after inhibiting the SIL1-Bip interaction, neuronal migration was significantly inhibited. In conclusion, the coordination function of SIL1 and Bip plays a key role in neuronal migration (Inaguma et al., 2014).

#### SIL1 and binding immunoglobulin protein jointly regulate reelin protein and affect development

In addition to regulating neuronal migration during cortical development, Reelin is currently believed to be involved in neuronal dendrite development and changes in brain plasticity (Niu et al., 2004; Jossin and Goffinet, 2007). The distribution of cortical neurons in heterozygous reeler (Reelin knockout) mice did not appear disordered, but their dendritic development was affected. Dendritic spine density decreased on the dendrites of pyramidal neurons in the CA1 region of the mouse hippocampus at 21 and 32 days after birth, whereas more mature neurons showed a lower density of dendritic spines accompanied by decreased expression levels of post-synaptic density protein 95 (PSD95) and NMDA receptors (Niu et al., 2008). These findings indicate that Reelin promotes dendritic development and the generation and maintenance of dendritic spines during post-natal development. The loss of Bip is one of the reasons for the down-regulation of Reelin protein, indicating that Bip may affect the development of dendrites and dendritic spines by affecting Reelin protein expression. Loss of SIL1, Bip, or Reelin function leads to similar abnormal phenotypes of neuronal migration (Leemhuis and Bock, 2011; Stranahan et al., 2013), and the loss of function of the ER molecular chaperone Bip inhibits normal Reelin expression. Thus, the relationship among these three proteins during neural development and whether the role played by Reelin protein in development is co-regulated by the coordination of Bip and SIL1 represent questions that deserve to be explored.

## The role of calnexin during neurodevelopment

Calnexin is a 90-kDa ER integral protein with a long amino-terminal domain (460 amino acids) located in the lumen of the ER, a hydrophobic *trans*-membrane domain and a short acidic cytoplasmic domain (91 amino acids). CNX binds to Ca^2+^ and acts as a chaperone in the transition of proteins from the ER to the outer membrane (Tjoelker et al., 1994). CNX and CRT constitute the CNX/CRT cycle and are responsible for the folding and quality control of newly synthesized proteins (Li et al., 2011).

Many researchers have confirmed the role of CNX as a molecular chaperone in neurodevelopment. The *CANX* gene (coding CNX) is activated in neuronal tissue early in embryonic development (Coe et al., 2008). Immunohistochemical staining of mouse brain tissue sections showed that CNX exists in hippocampal neurons and is expressed at high levels in soma, dendrites, and synapses (Itakura et al., 2013). Additional studies have shown that in mice, CNX deficiency leads to a phenotype of peripheral axonal demyelination and reduced peripheral nerve conduction velocity, demonstrating that CNX may play an essential role in neuron growth and development (Jung et al., 2018).

### Calnexin regulates dendrite and synapse development

Denzel et al. found that approximately 50% of CNX knockout mice died within 48 h after birth, most of the remaining mice died within 4 weeks, and only a few mice survived to 3 months (Denzel et al., 2002). CNX gene knockout (*cnx*^–/–^) mice exhibited significant movement impairment, including unsteady gait, inactive walking, and obvious trunk ataxia. The authors then examined the sciatic nerve in the gene-deficient mice through electron microscopy and found that most of the mice exhibited a reduced number of medullary nerve fibers and a reduced sciatic nerve diameter (Denzel et al., 2002). However, the cause of the short-lived death of *cnx*^–/–^ mice has not yet been discovered but might be related to the coordinating role of CNX proteins in development. This hypothesis deserves further exploration.

Recent studies assessing the localization of CNX and NMDA receptors on neurons based on specific immunostaining and biotin chemical reagent labeling revealed that a considerable portion of CNX was localized on the cell surface of cultured neurons, and this localization was regulated by NMDA receptors (Itakura et al., 2013). These results suggest that CNX may play an essential role in NMDA receptor-mediated neuronal function. Other studies demonstrated that α-amino-3-hydroxy-5-methyl-4-isoxazole-propionic acid (AMPA) receptors interact with CNX and Bip in neuronal cell bodies and hippocampal pyramidal neuron dendrites (Rubio and Wenthold, 1999; Gerrow and Triller, 2010). In addition, the AMPA receptor is a tetramer composed of four subunits, GluA1, GluA2, GluA3, and GluA4. This receptor is expressed in both neurons and glial cells (Pick and Ziff, 2018). It is well known that NMDA receptors and AMPA receptors play essential roles in central nervous system development. NMDA receptors are involved in experience-dependent synapse and dendritic development (Nadarajah and Parnavelas, 2002; Luo et al., 2006), whereas AMPA receptor expression directly affects neuronal dendritic complexity, synaptic maturation, and neural network formation (Buffington et al., 2014). Defects in AMPA receptor expression are the underlying causes of developmental diseases, degenerative diseases, and cognitive dysfunction (Kessels and Malinow, 2009; Henley and Wilkinson, 2016; Guo and Ma, 2021). However, the specific effects of CNX loss on NMDA receptor or AMPA receptor membrane expression or function remain to be investigated. Moreover, studies have shown that CNX can be attached to membranes through NMDA receptor-mediated pathways (Itakura et al., 2013). Therefore, CNX may regulate central development by participating in receptor function at the cell membrane. In addition, CNX regulates calcium levels in the ER (Roderick et al., 2000), which subsequently regulates receptor transport to influence synaptic strength (Turrigiano, 2008). Therefore, CNX may directly affect synaptic strength and synaptic development through this mechanism. However, the direct correlation between CNX and synaptic development and function needs to be expanded.

### Calnexin cooperates with the cytoskeleton-associated protein to regulate neural plasticity

Activity-regulated cytoskeleton-associated (Arc) protein is a master regulator of long-term synaptic plasticity and memory formation (Bramham et al., 2010; Korb and Finkbeiner, 2011; Shepherd and Bear, 2011; Nikolaienko et al., 2017). Arc proteins control synaptic strength by promoting AMPA receptor endocytosis (DaSilva et al., 2016) and activity-dependent long-term changes in synaptic strength (Ehlers, 2000; Newpher and Ehlers, 2008). Arc overexpression decreased the density of GluA1 subunits of AMPA receptors on the cell surface and AMPA receptor-mediated miniature excitatory post-synaptic currents (mEPSCs). Arc mutations that prevent clathrin-adaptor protein 2 (AP-2) interaction reduce Arc-mediated endocytosis of GluA1 and abrogate AMPA receptor-mediated mEPSCs reduction (DaSilva et al., 2016). Endocytosis is the process by which cells internalize substances through the plasma membrane. Endocytosis plays an important role in different stages of the development of the nervous system, such as the formation of continuous synaptic transmission (Sudhof, 2004), the entry of drugs into the central nervous system (Smith et al., 2008), and the internalization of receptor-ligand complexes to nerve endings for intracellular signaling (Gloor et al., 2001). CNX is also involved in clathrin-mediated endocytosis of neurons (Li et al., 2011). Kraus et al. performed an electron microscopy analysis of the cerebellum of 7-day-old wild-type and CNX-null mice to assess whether CNX is involved in neuronal endocytosis. The results showed that the number of synaptic vesicles was significantly increased in the cerebellum of CNX-deficient mice compared with wild-type mice, demonstrating that CNX deficiency leads to increased endocytic activity in the mouse neuronal system (Kraus et al., 2010). Recent studies have found a direct interaction between Arc and CNX in neurons, and the interaction between recombinantly expressed glutathione S-transferase (GST)-tagged Arc and endogenous CNX has also been found in HEK293, SH-SY5Y neuroblastoma, and PC12 cells (Myrum et al., 2017). Given that both Arc and CNX mediate clathrin-dependent endocytosis and that both are expressed in excitatory synapses, it is possible that Arc and CNX cooperate in regulating endocytosis. Therefore, whether CNX is involved in the synaptic effect is also a mechanism that needs further exploration.

## Calreticulin regulates neurodevelopment

Calreticulin is a multifunctional protein with a molecular weight of 46 kDa. CRT is expressed in almost all eukaryotic cells and is preferentially located in the ER. Depending on the sublocalization of CRT in cells, CRT has different physiological functions, including chaperone function and lectin binding. CRT was initially considered to be a high-affinity calcium-binding protein (Rauch et al., 2000), and early studies focused on its role as a calcium storage molecule in the ER (Michalak et al., 1992), including regulating calcium channel activation and maintaining intracellular calcium homeostasis (Chen et al., 2015). Later, CRT was considered a multifunctional protein that interacts with other proteins and participates in various processes (Michalak et al., 1999). In recent years, the focus of CRT research has been refocused to its role as an ER chaperone that aids in glycoprotein folding (Pacheco et al., 2020). The role of CRT in neurodevelopment mainly involves regulating embryonic development, neural differentiation, and axonal growth in the peripheral nervous system.

### Calreticulin regulates embryonic development of the central nervous system

To study the role of CRT during development *in vivo*, Rauch et al. generated CRT-deficient mice by targeted inactivation of the CRT gene. The results demonstrated that CRT heterozygous mice were viable and fertile, whereas *crt*^–/–^ mice were embryonic lethal and exhibited multiple disease phenotypes, including cardiomyopathy, anencephaly, and omphalocele (Rauch et al., 2000). In addition, CRT is also important for cranial neural tube closure and is highly expressed in the developing brain during neurogenesis (Mesaeli et al., 1999). Neural tube formation is a complex process that requires the interaction of neuroepithelial cells and the underlying mesenchyme (Lynch et al., 2012; Correll et al., 2019). Disruption of neural tube closure was found in several CRT knockout mice, in which proteins involved in neuronal migration and calcium signaling were inactivated (Solheim et al., 1997; Rauch et al., 2000). These results demonstrate that CRT is important for embryonic brain development, especially the neural tube development. However, it is currently unclear which proteins interact with CRT to regulate embryonic neural development.

### Calreticulin regulates neuronal differentiation

Neurotrophic factors, including nerve growth factor (NGF), brain-derived neurotrophic factor (BDNF), neurotrophic factor-3 (NT-3), and neurotrophic factor-4/5 (NT-4/5), are key tissue factors that control nervous system development (Chao, 2003). The NGF/tyrosine receptor kinase A (TrkA) signaling pathway is necessary for neural development, and abnormalities in this pathway can lead to abnormal neuronal differentiation. Shih et al. found that in NGF-stimulated PC12 cell differentiation, CRT levels were increased by activation of mitogen-activated protein kinase (MAPK) mediated by extracellular signal-regulated kinase (ERK). CRT deficiency significantly reduced NGF-induced neuronal differentiation. Furthermore, CRT overexpression enhanced neuronal differentiation through simultaneous activation of the ERK-dependent MAPK pathway (Shih et al., 2012). The Ca^2+^ regulation ability of CRT is also essential for NGF-triggered neuronal differentiation (Pilquil et al., 2020). In addition, studies have shown that CRT is a novel oncogenic N-MYC (MYCN) inhibitor that can down-regulate MYCN promoter activity and protein expression to regulate neuronal differentiation. This study also found that CRT-mediated MYCN inhibition resulted in increased neurite length (Lee et al., 2019).

### Calreticulin regulates axonal growth in the peripheral nervous system

Calreticulin localizes to axons of cultured dorsal root ganglion neurons *in vitro* and peripheral nervous system axons *in vivo* (Willis et al., 2005; Vuppalanchi et al., 2010), and automatic axonal regeneration occurs after injury to adult peripheral neurons (Bradke et al., 2012). The intrinsic growth capacity of peripheral nervous system axons after injury is greater than that of central nervous system axons. Pacheco et al. constructed *in vitro* and *in vivo* neuronal axonal injury models to study the relationship between CRT and axonal regeneration after axonal injury. Intra-axonal CRT levels were elevated after peripheral nerve injury *in vivo*. Unilateral sciatic nerve crush injury was performed in the mid-thigh of male SD rats, and then sciatic nerve sections were collected and stained 1 h, 6 h, 18 h, and 7 days after the crush injury. Compared with the control sciatic nerve, axonal CRT levels were significantly increased 6 h after injury and reached a maximum at 18 h after injury. The authors then performed axotomy on dorsal root ganglion neurons to study the effect of CRT deletion on the post-operative axonal growth process. The results showed that the shRNA-mediated CRT deletion group exhibited exacerbated post-axotomy contractions. In contrast, axon-targeted expression of unrestricted CRT mRNA reduces contraction and promotes axon regeneration after axotomy *in vitro* (Pacheco et al., 2020). In conclusion, the overexpression of axon-targeted CRT promotes axon regeneration after axonal injury, indicating that CRT plays an important role in axonal growth.

## The role of protein disulfide isomerase in neurodevelopment

Protein disulfide isomerase is a class of multifunctional proteins present in the ER lumen, including endoplasmic reticulum protein 57 (ERp57, also known as PDIA3), endoplasmic reticulum protein 29 (ERp29), protein disulfide isomerase-1 (PDI-1), endoplasmic reticulum protein 72 (ERp72), protein disulfide isomerase A1 (PDIA1), and protein disulfide isomerase p (PDI p) (Turano et al., 2002). As a molecular chaperone, PDI binds to the stretched or partially folded peptide chain through its polypeptide binding site to form an intermediate to prevent incorrect protein folding (Wilkinson and Gilbert, 2004).

### Protein disulfide isomerase-1 regulates neuronal migration

Protein disulfide isomerase-1 controls neuronal migration by regulating Wnt secretion. PDI is a family of protein chaperones that reside in the ER and are capable of catalyzing the formation (oxidation), cleavage (reduction), and rearrangement (isomerization) of disulfide bonds (Wilkinson and Gilbert, 2004). The formation of a proper Wnt protein structure requires the formation of disulfide bonds, which are critical for Wnt secretion and signaling (Zhang X. et al., 2012; MacDonald et al., 2014). Wnt proteins play essential roles in neuronal migration and axon-dendritic guidance in vertebrates (Yoshikawa et al., 2003; Bocchi et al., 2017), and they also control neural developmental processes in *C. elegans*, including neuronal migration, polarity, and axon guidance (Coudreuse et al., 2006; Hilliard and Bargmann, 2006; Pan et al., 2006). Therefore, abnormal Wnt expression or function significantly impairs neural development.

PDI-1 controls the secretion of EGL-20, the Wnt homolog in *C. elegans*, and therefore affects neuron migration (Torpe et al., 2019). EGL-20/Wnt is expressed and secreted in subcutaneous (epidermal) and muscle cell subsets of *C. elegans* (Whangbo and Kenyon, 1999). EGL-20 function is critical for the migration of hermaphroditism-specific neuron (HSN) (Desai et al., 1988). Torpe et al. used HSN development as a readout of EGL-20 function to identify molecules that control Wnt maturation and secretion. They hypothesized that PDI is important for EGL-20-directed HSN development. The expression of five PDI-encoding genes (pdi-1, pdi-2, pdi-3, pdi-6, and C14B9.2) in *C. elegans* was reduced using RNA interference, and the development of HSN was analyzed. This study found a specific requirement for PDI-1 during HSN development with approximately 20% of PDI-1-silenced animals exhibiting a phenotype deficient in HSN migration. Subsequently, to determine whether PDI-1 also controls EGL-20-dependent post-embryonic neuron development, the authors performed post-embryonic neuronal migration assays in PDI-1-deficient animals and found that PDI-1 knockout animals exhibited migration defects (Torpe et al., 2019). PDI inhibitors reduce Wnt3a secretion in human cells. Most mammalian genomes contain 19 Wnts and greater than 20 PDI proteins, which exhibit distinct expression domains and biological functions (Ellgaard and Ruddock, 2005; Galligan and Petersen, 2012; Willert and Nusse, 2012). Mammalian Wnt3a secretion was abolished when a single cysteine residue was replaced by alanine (MacDonald et al., 2014), suggesting that Wnt secretion is influenced by disulfide coordination. These results suggest that PDI can affect neuronal migration by regulating the secretion of Wnt and its homolog EGL-20.

The role of the Wnt signaling pathway in early cortical development is not limited to the migratory localization of neurons. It is also involved in neurogenesis, neuronal differentiation, and axon-dendritic guidance (Freese et al., 2010; Munji et al., 2011). In addition, the atypical Wnt signaling pathway is involved in the assembly of neural circuits (Hinata et al., 2007; Wang et al., 2011), especially dendrite development and synaptogenesis in cultured pyramidal neurons (Salinas and Zou, 2008). Wnt pathway dys-regulation is closely related to human neurological diseases (Castelo-Branco et al., 2003). However, the mechanism by which PDI further affects other developmental processes by affecting the Wnt signaling pathway is unclear and deserves further exploration.

## The role of sigma-1 receptor in neurodevelopment

Sigma-1 receptor is a 223-amino acid long *trans*-membrane ER chaperone with a molecular weight of 25.3 kDa (Hanner et al., 1996; Dalwadi et al., 2022). Most Sig-1R proteins are located in the ER, especially in the mitochondria-associated ER membrane, and are critical for regulating energy balance and calcium homeostasis (Hayashi and Su, 2007). Sig-1R is highly expressed in the central nervous system, especially in the cortex, basal ganglia, spinal cord, and brainstem. It is also vital to maintain the proper functioning of motor neurons (Su et al., 2010). Sig-1R has recently attracted extensive attention as a potential drug target for neurological diseases and cancer (Merlos et al., 2017; Penke et al., 2018).

Recently, Sig-1R was shown to be important in neurodevelopmental research as an ER chaperone and is known to be involved in learning and memory. Within the nervous system, Sig-1R is predominantly localized in the gray matter of neurons and multiple glial cell types (Peviani et al., 2014; Robson et al., 2014). In addition to its role in regulating intercellular signaling (Su et al., 2010), neuroprotection (Ruscher et al., 2011), neural recovery (Ruscher et al., 2012), neuroplasticity (Kourrich et al., 2012), and neurotransmitter release (Zheng, 2009), Sig-1R also regulates various neural developmental processes, such as hippocampal neuronal dendritic morphogenesis, dendritic spine maturation, and neuroplasticity.

### Sigma-1 receptor regulates neuronal morphogenesis

Sigma-1 receptor regulates neurite outgrowth (Ishima et al., 2014) as well as primary neuron morphogenesis (Ishima and Hashimoto, 2012). Dendritic spines in hippocampal neurons play important roles in neuroplasticity and memory formation. siRNAs were used to silence the expression of Sig-1R to study their role in the morphogenesis of primary hippocampal neurons *in vitro*. The results showed impaired dendrite extension and branching after Sig-1R gene silencing. Moreover, Sig-1Rs also have a major impact on the formation and maturation of dendritic spines in later stages (Tsai et al., 2009). In rat hippocampal primary neurons, reducing the expression of Sig-1Rs by siRNA resulted in defects in dendritic spine formation. Tsai et al. observed and analyzed the dendritic spine morphology and maturity of cultured hippocampal neurons. On DIV 16, the control group neurons formed filamentous protrusions on the dendrites. On DIV 22, control neurons formed clusters of short, thick mushroom-like spine heads without filopodia, indicating spine maturation. In contrast, Sig-1R-siRNA-transfected (siSig-1R-tf) neurons formed elongated protrusions with a few spine heads at the tips. The longer the culture time, the more pronounced the abnormal phenotype. This finding suggests that Sig-1Rs are endogenous regulators of hippocampal dendritic spine formation (Tsai et al., 2009).

### Sigma-1 receptor regulates synaptic function

Sigma-1 receptor also plays an important role in the development of synaptic function (Ryskamp et al., 2019). Active synapses express specific proteins and receptors for proper function. Studies have shown that Sig-1R knockdown inhibits functional synapse formation. GluA2, GluA3, and PSD-95 staining revealed a strong immune response on dendritic spines in control neurons. However, this response was not observed in filopodia protrusions of siSig-1R-tf neurons, whereas these proteins were still present in dendritic shafts. The axonal terminals of siSig-1R-tf neurons also showed significantly reduced immune responses to synaptophysin. Neurons labeled with FM4-64, a membrane-selective red fluorescent dye, were depolarized with KCl to measure synaptic activity. The results showed that siSig-1R-tf neurons did not form functional synapses. These results suggest that Sig-1Rs have essential effects on synapse formation and synaptic function (Ryskamp et al., 2019).

Earlier, we mentioned that the NMDA receptor is associated with learning and memory. A study found that changes in Sig-1R can cause changes in NMDA receptor expression. Sig-1R was overexpressed by intraperitoneal injection of the Sig-1R activation agonist SKF10,047 in SD rats, and subsequent immunofluorescence staining revealed increased GluN2A, GluN2B, and PSD95 expression (de Montigny et al., 1992; Monnet et al., 1994, 1996). This effect was abolished after the addition of the Sig-1R antagonist BD1063 (Matsumoto et al., 1995; McCracken et al., 1999). In addition, they found increased interactions between Sig-1R and GluN2 subunits as well as increased surface levels of NMDA receptor upon Sig-1R activation, which suggests that Sig-1R regulates the expression of NMDA receptor and related neural function (Pabba et al., 2014).

### Sigma-1 receptor regulates mitochondrion-associated endoplasmic reticulum membrane conduction and affects neural development

The membrane of the ER of a cell forms contacts directly with mitochondria, and the contact is referred to as the mitochondrion-associated ER membrane (MAM) (Mori et al., 2013). The MAM is a subdomain of the ER that engages in a direct physical association with mitochondria (Rizzuto et al., 2004; Csordás et al., 2006; Szabadkai et al., 2006). The MAM integrates many signaling pathways and is important for cellular survival because it serves as the “tunnel” for lipid transport and Ca^2+^ signaling between the ER and mitochondria (Cárdenas et al., 2010). Sig-1Rs are ER chaperones that localize specifically at the MAM and regulate a variety of cellular functions including Ca^2+^ signaling between ER and mitochondria, neuronal differentiation, ion channel activities, and notably cellular survival (Aydar et al., 2002; Fontanilla et al., 2009; Fukunaga and Moriguchi, 2017). However, exactly how Sig-1R regulates the neuronal differentiation process at the MAM is not fully clarified. Neurons are highly differentiated cells and require great amounts of ATP for the maintenance of cell membrane ionic gradients and neurotransmission (Xavier et al., 2016). Mitochondria, the main intracellular energy transducers, play a key role in neural development by producing ATP and biosynthetic substrates, regulating Ca^2+^ homeostasis, and initiating apoptosis (Kann and Kovács, 2007). Given the vital role of Sig-1R in the MAM and the importance of energy transport for neural development, exploring the energy transduction mechanism of Sig-1R in the MAM may be key to studying the developmental process.

## The function of prefoldin during neurodevelopment

Prefoldin is a heterohexameric protein complex (Glover and Clark, 2015) consisting of two α subunits (PFDN3 and PFDN5) and four β subunits (PFDN1, PFDN2, PFDN4, and PFDN6). PFDN interacts with nascent polypeptide chains and can act as a substitute for Hsp70 *in vitro* to facilitate protein folding and prevent intermolecular aggregation (Siegert et al., 2000). In recent years, studies have found that PFDN regulates the toxicity of misfolded proteins, including those that lead to neurodegenerative diseases. For example, PFDN can protect neuronal cells from polyglutamine toxicity by preventing aggregate formation or maintaining proteostasis by unfolding misfolded proteins in physiopathological conditions (Tashiro et al., 2013; Tahmaz et al., 2022). PFDN-deficient mice exhibit phenotypic features of defective cytoskeletal function, including ciliary dyskinesia, neuronal loss, and defects in B- and T-cell development and function. As ubiquitous components of the cytoskeleton, actin and tubulin play essential roles in multiple stages of neural development, including dendritic axon growth, neuronal polarization, neuronal migration, and synaptic signal transduction (Cao et al., 2008; Bertling and Hotulainen, 2017), whereas efficient synthesis of actin and tubulin requires the participation of the protein complex formed by PFDN and t-complex protein 1 ring complex (TRiC)/chaperonin-containing t-complex protein 1 (CCT). As an ER chaperone, PFDN promotes the post-translational folding of actin and other cytoskeletal proteins through the t-complex protein 1 (TCP1)-containing loop complex chaperone TRiC (Cao et al., 2008). PFDN and TCP1 play essential roles in metazoan development. In *C. elegans*, RNA interference screens revealed that knockdown of a single PFDN or TCP1 subunit resulted in morphological abnormalities and distinct penetrating embryonic lethal phenotypes (Delgehyr et al., 2012), suggesting that these proteins are essential in multiple tissue development and early embryonic development (Monzo et al., 2010). In contrast, PFDN1-deficient mice develop defective cytoskeletal function, loss of neural tracts, hydrocephalus, neuromuscular defects, abnormal lymphocyte development and function, and shortened lifespan (Lee et al., 2011). Furthermore, abnormal actin and/or tubulin cytoskeleton assembly and microtubule formation may underlie the *Pfdn5^nmf^*^5*a*^ (encoding PNDF5) disease phenotype, including photoreceptor degeneration (Kwon et al., 2019), central nervous system abnormalities, and male sterility (Lee et al., 2011). Although PFDN5 is expressed in various tissues, defects in PFDN5 homozygotes appear to be limited to neuronal cells. Genetic disruption of the mouse *Pfdn5* gene results in reduced formation of microtubules and microfilaments, leading to progressive neurodegeneration, hydrocephalus, and reproductive abnormalities. These results suggest that PFDN5 is required for normal sensory and neuronal development (Lee et al., 2011).

## Endoplasmic reticulum molecular chaperones and neurodevelopmental disorders

Neurodevelopmental disorders (NDDs) refer to the abnormal development of the nervous system caused by inherited or acquired diseases, resulting in brain dysfunction. NDDs include intellectual disability (ID), autism spectrum disorder (ASD), attention-deficit hyperactivity disorder (ADHD), and bipolar disorder (BD) (Rutter et al., 2006; Thapar et al., 2017; Ismail and Shapiro, 2019; Morris-Rosendahl and Crocq, 2020). At present, the global prevalence of unexplained ID is 2–3% (Olusanya et al., 2020), the prevalence of ASD is 1–2% (Baxter et al., 2015; Zablotsky et al., 2015), the prevalence of ADHD is 5–7.2% (Polanczyk et al., 2007, 2014; Sokolova et al., 2017; Wang T. et al., 2017), and the prevalence of BD is 1–4% (Merikangas et al., 2011). Numerous studies have shown that children with NDDs have a high incidence of mental health problems compared with normal children (Arim et al., 2015; Eyre et al., 2019). NDDs bring heavy economic and mental burdens to families and society, seriously affect the quality of life of patients and their families and represent a public health problem of high concern worldwide. The pathogenesis of NDDs is complex. Environmental risk factors, such as the inflammatory response, immune disorders and metabolic disorders, and genetic factors, such as neuronal dysfunction and chromosome deletion, are involved in the occurrence of NDDs (Bădescu et al., 2016). The abnormal expression of ER chaperone proteins can lead to the dys-regulation of neurodevelopmental processes and participate in the occurrence of many neurodevelopmental diseases.

### Endoplasmic reticulum molecular chaperones and autism spectrum disorder

Autism spectrum disorder is a brain developmental disorder characterized by language disorders, social difficulties, and repetitive and stereotyped behaviors (Lai et al., 2014).

#### Calreticulin and autism spectrum disorder

Current studies have reported copy number variations (CNVs) in specific genes, and the ASD-related literature has cited the involvement of ER chaperone proteins. However, the molecular mechanisms are not well understood. For example, when studying the relationship among CD47 overexpression, brain overgrowth and 16p11.2 deletion syndrome, Li et al. found that ER chaperone CRT-mediated phagocytosis was related to the pathogenesis of ASD (Li et al., 2021). CNV at 16p11.2 has been implicated in neuropsychiatric disorders, such as ASD and schizophrenia (Kumar et al., 2008; Weiss et al., 2008; McCarthy et al., 2009; Zarrei et al., 2019). Carriers with 16p11.2 deletions tend to have macrocephaly (or enlarged brain), whereas carriers with 16p11.2 duplications often have microcephaly (Bartholomeusz et al., 2002; Hazlett et al., 2011; Maillard et al., 2015; Sacco et al., 2015). Human induced pluripotent stem cells (hiPSCs) from controls and subjects with 16p11.2 deletions and duplications were employed to understand the underlying mechanisms regulating brain overgrowth. CD47 (the “do not eat me” signal) was found to be overexpressed in cells with the 16p11.2 deletion vector, and CRT was also highly expressed. Cells with high CRT expression should be eliminated by immunophagocytosis, whereas cells escape immunophagocytosis due to high expression of CD47 (Oldenborg et al., 2000). The phagocytosis and CRT expression of hiPSCs with 16p11.2 deletion were restored to the control level when anti-CD47, a CD47 blocking agent, was applied (Li et al., 2021). This finding indicates that the normal phagocytosis mediated by CRT is affected in 16p11.2 null cells, and this mechanism may be involved in the pathogenesis of ASD.

#### Protein disulfide isomerase A1 and autism spectrum disorder

Lammert et al. found that increased PDIA1 was associated with ASD (Lammert et al., 2017). Studies have shown that RELN gene mutation can lead to ASD complications (Fatemi et al., 2005). In the ASD mouse model based on the RELN R2290C (Reelin mutant gene) (Iossifov et al., 2014; Wang et al., 2014; Lammert and Howell, 2016) mutation, PDIA1 expression was increased in the neurospheres of RELN R2290C heterozygous (+/-) as well as in the cerebellum of RELN Orleans (Orl) +/- mice. Reelin protein is highly expressed in the developing cerebral cortex and cerebellum and plays an important role in neuronal migration. As an ER-resident chaperone, PDIA1 can ensure the formation of correct disulfide bonds in nascent proteins (Parakh and Atkin, 2015), but its overexpression may cause neuropathological diseases (Perri et al., 2016; Zeeshan et al., 2016). This finding indicates that the increased expression of PDIA1 may be associated with the risk of ASD.

#### Sigma-1 receptor and autism spectrum disorder

Fragile X syndrome (FXS) is a disorder of synaptic development and dysfunction (Hagerman et al., 2009) and is the most prevalent genetic form of ID, ASD, and ADHD (Boyle and Kaufmann, 2010; Budimirovic and Kaufmann, 2011; Kaufmann et al., 2017). FXS mouse models recapitulate anxiety phenotypes similar to those observed in the clinic. Blarcamesine is a Sig-1R agonist (Villard et al., 2011), and the important role of Sig-1R in calcium homeostasis and synaptic function (Su et al., 2016; Schmidt and Kruse, 2019) makes blarcamesine a potential candidate for FXS. Blarcamesine has shown preliminary efficacy in patients with Alzheimer’s disease, Rett syndrome (RTT), synaptic neurodegeneration and NDDs (Hampel et al., 2020). Reyes et al. conducted research on the role of blarcamesine in FXS (Reyes et al., 2021). These researchers used the Fmr1 knockout FXS mouse model (Bakker et al., 1994; Mientjes et al., 2006; Dahlhaus, 2018) to evaluate the effect of blarcamesine on key cognitive and behavioral aspects that represent the FXS phenotype. When FXS mice were administered blarcamesine, two key neurobehavioral phenotypes, the open field test (hyperactivity) and contextual fear conditioning (associative learning), returned to normal.

Kaufmann et al. conducted further studies on the role of blarcamesine in RTT (Kaufmann et al., 2019). RTT is a progressive, non-inherited, X-linked neurodevelopmental disorder with an incidence of approximately 1 in 10,000 female births (Laurvick et al., 2006; Neul et al., 2010). Most patients carry a mutation in the methyl-CpG-binding protein 2 (*MECP2*) gene, which encodes a rich transcriptional regulator in the brain (Amir et al., 1999; Neul et al., 2014). The disorder is characterized by a variety of neurological impairments, particularly affecting cognition (i.e., developmental delay, ID, communication deficits), behavior, and motor and autonomic dysfunction (Neul et al., 2010; Kaufmann et al., 2016). No specific effective treatment is available for RTT, and treatment is mainly symptomatic. Using female Mecp2 mutant mice (Samaco et al., 2013; Lombardi et al., 2015; Tan and Zoghbi, 2019) as the research object, the authors and others administered the Sig-1R agonist blarcamesine and found that motor defects, acoustic starvation defects and visual defects were significantly improved. These data suggest that Sig-1Rs play an important role in FXS and in neurological disorders that are genetically characterized as FXS.

### Endoplasmic reticulum molecular chaperones and intellectual disability

Intellectual disability (ID) is a common disorder of nervous system development that leads to intellectual disabilities. Various factors can cause ID, including loss of important synaptic function, abnormal neuronal morphological development, and neuronal migration and localization disorders (Kaufman et al., 2010; Ropers, 2010; Mehregan et al., 2016).

#### SIL1 and intellectual disability

Studies have shown that *SIL1* gene mutations causes Marinesco-Sjögren syndrome (MSS), and it is the only gene that has been found to cause MSS. MSS is a rare autosomal recessive genetic disease. Clinical studies have found that approximately 90% of patients with MSS caused by *SIL1* mutation present with moderate to severe ID, motor delay, congenital cataract, cerebellar atrophy, and other NDDs (Roos et al., 2014; Osborn et al., 2017). It has been well established that the decreased interaction between Bip and SIL1 or the loss of SIL1 function alone leads to the abnormal migration and localization of cortical neurons, representing a possible cause of ID (Krieger et al., 2013; Inaguma et al., 2014).

#### Calnexin and intellectual disability

In a study of mucopolysaccharidosis type II (MPS II), Osaki et al. demonstrated that CNX expression directly affects intellectual deficits caused by iduronate 2-sulfatase (IDS) mutations (Osaki et al., 2019). MPS II is one of the most common mucopolysaccharidoses (Wraith et al., 1987; Stapleton et al., 2018) and is caused by mutations in the gene encoding IDS (Wilson et al., 1990). The loss of IDS function leads to the accumulation of heparan sulfate and dermatan sulfate of glycosaminoglycans throughout the body, resulting in skeletal deformities, retardation, rigid joints, and thick skin (Ficicioglu et al., 2018). It was found that mutant IDS retained in the ER requires binding to CNX to achieve folding. Thus, CNX knockdown reduces the transport of mutant IDS from the ER to lysosome and its enzymatic activity. These studies indicate that proper folding by interaction with CNX ensures its functional activity. These findings reveal the existence of a role between IDS and CNX for the treatment of ID caused by MPSII and IDS mutations (Osaki et al., 2019).

#### Sigma-1 receptor and intellectual disability

α-thalassemia X-linked intellectual disability (ATR-X) syndrome is caused by mutations in *ATRX* (Gibbons et al., 1992, 1995). ATR-X syndrome is characterized by a variety of clinical manifestations, including severe ID, facial dysmorphism, genital abnormalities, and seizures (Gibbons et al., 2008). Similarly, ATR-X model mice lacking *Atrx exon* 2 (Nogami et al., 2011) show phenotypes similar to the symptoms of ID noted in humans, cognitive deficits (Kaufmann and Moser, 2000; Levenga and Willemsen, 2012), and abnormal dendritic formation (Shioda et al., 2011). Yamaguchi et al. investigated whether Sig-1R promotes the increased activity of neurotrophic factors, such as BDNF, to induce neuroprotection and nerve regeneration (Yamaguchi et al., 2018). They found that treatment with the Sig-1R activator SA4503 (Matsuno et al., 1996) reversed axonal development and dendritic spine abnormalities in primary cultured cortical neurons of ATR-X model mice. In addition, SA4503 treatment rescued the cognitive deficits noted in ATR-X model mice.

#### Protein disulfide isomerase family A member 3 and intellectual disability

Recessive gene mutations underlie many developmental disorders and often lead to disabling neurological problems (Martin et al., 2018). Bilches et al. conducted clinical and genetic studies on large close relatives with ID (Bilches Medinas et al., 2022). These researchers isolated a homozygous mutant of PDIA3, c.170G > A (p. Cys57Tyr or C57Y), from the genes of clinical patients with ID and determined that this disease was associated with PDIA3. PDIA3 is an oxidoreductase containing a thioredoxin-like domain that catalyzes the formation and isomerization of disulfide bonds in the ER and is associated with syndromic ID (Ellgaard and Ruddock, 2005). Experiments in zebrafish embryos demonstrated that the PDIA3*^C^*^57*Y*^ mutation is pathogenic and causes developmental defects, such as axonal disorganization and skeletal abnormalities. Abnormal PDIA3*^C^*^57*Y*^ expression in the hippocampus leads to impaired synaptic plasticity and memory consolidation (Bilches Medinas et al., 2022).

### Endoplasmic reticulum molecular chaperones and bipolar disorder

Bipolar disorder is a common mental disorder characterized by recurrent episodes of mania, hypomania, and depression. BD is characterized by mood swings between elevated and depressed moods (Rosso et al., 2007). The exact mechanism of these mood swings remains unclear and needs to be further elucidated.

#### Binding immunoglobulin protein and bipolar disorder

There is increasing evidence from previous *in vitro* studies that the ER, as a protein folding factory, plays a major role in BD (So et al., 2007; Hayashi et al., 2009; Pfaffenseller et al., 2014). The role of the ER molecular chaperone Bip in BD diseases is more reflected in ER stress. ER stress activates the UPR (Zhang and Kaufman, 2008; van Schadewijk et al., 2012), which is partially impaired in BD (Pfaffenseller et al., 2014). Therefore, Bengesser et al. analyzed *Bip* and C/EBP homologous protein (*CHOP*) gene expression and X-box binding protein 1 (*XBP1*) splicing in the peripheral blood of BD patients and control study participants (Bengesser et al., 2018). They isolated RNA from fasting blood of BD patients and control study participants, reverse transcribed it into cDNA, and analyzed *Bip* and *CHOP* gene expression. The results significantly increased *Bip* gene expression in BD samples. In addition, valproate, an atypical antipsychotic drug, has been shown to play a neuroprotective role by increasing the expression of chaperone proteins that assist ER protein folding, such as CRT and Bip (Chen et al., 2000; Bown et al., 2002; Kim et al., 2005). This neuroprotective effect has been suggested to be potentially related to the pathophysiology of neuropsychiatric disorders, such as schizophrenia and BD (Aghajani et al., 2006). However, the role of the specific pathophysiology of this neurological disorder remains to be elucidated.

### Endoplasmic reticulum molecular chaperones and attention-deficit hyperactivity disorder

Attention-deficit hyperactivity disorder is a syndrome characterized by inattention, hyperactivity, emotional impulsivity, and learning difficulties (Tripp and Wickens, 2009).

At present, there are few studies on the role of molecular chaperones in ADHD, and more relevant studies exist at the level of drug treatment targets. Methylphenidate (MPH) is a commonly used stimulant drug for ADHD (Capp et al., 2005; Arnsten, 2006). The symptoms of ADHD patients are mostly consistent with dysfunction in the pre-frontal cortex (PFC) (Barkley et al., 1992), a highly functional region that directs and organizes attention, thoughts, and emotions (Arnsten and Li, 2005). It has been demonstrated that low-dose MPH injection into the PFC can improve working memory performance (Arnsten and Dudley, 2005). High MPH doses can cause behavioral sensitization and a high risk of addiction (Amini et al., 2004). Sig-1R receptor proteins are highly distributed in the PFC, striatum and hippocampus. At the cellular level, Sig-1R is post-synaptic (Hayashi and Su, 2004) and is an excitatory target for the treatment of ADHD (e.g., cocaine) (Matsumoto et al., 2001; Maurice et al., 2002). MPH was found to increase NMDA receptor-mediated synaptic transmission, which was mediated by Sig-1R. Under the same dose of MPH stimulation, the NMDA-induced post-synaptic strength in the hippocampus of mice supplemented with the Sig-1R agonist PRE-084 was significantly higher than that of the control group (Motawe et al., 2020). Similarly, pretreatment with the Sig-1R antagonist BD1063 effectively prevented hyperactivity caused by MPH hyperactivity (Matsumoto et al., 1995), confirming the importance of Sig-1R receptors in excitatory synaptic transmission (Zhang C. L. et al., 2012). Therefore, the active use of Sig-1R receptor ligands in the treatment of ADHD and prevention of MPH-induced addiction may have unexpected effects.

## Conclusion

**FIGURE 1:**
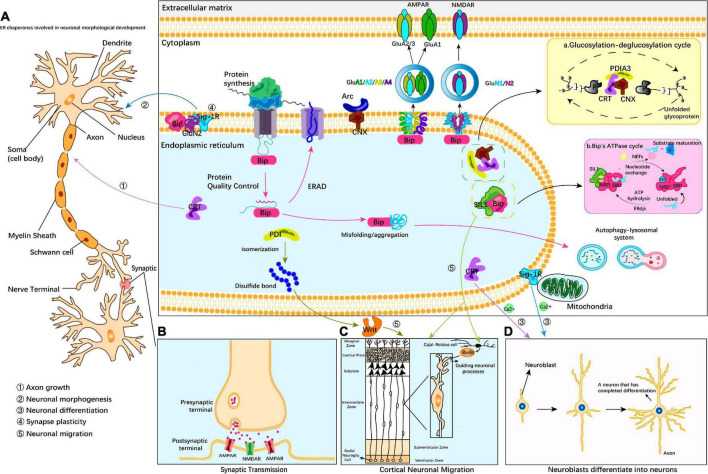
Endoplasmic reticulum (ER) molecular chaperones involved in neurodevelopment. ER molecular chaperones regulate central nervous development through two mechanisms: 1. ER chaperones directly act on specific developmental periods and regulate developmental stages. 2. ER chaperones indirectly act on secretory proteins or receptor proteins and regulate ER stress by ensuring protein homeostasis. CRT and Sig-1R regulate neuronal morphogenesis and dendritic axon growth **(A)**; Bip, SIL1, and PDI-1 regulate cortical neuron migration **(C)**; CRT regulates Ca^2+^ to guide NGF differentiation into mature neurons **(D)**; Bip, CNX, and Sig-1R regulate synaptic function **(B)**. Indirect regulation mainly affects the secreted protein Reelin and AMPA and NMDA membrane receptors **(C)**, which act on the post-synaptic membrane and nerve migration. Bip proteins are involved in endoplasmic quality control and transport misfolded proteins for degradation, thereby maintaining protein homeostasis under ER stress conditions. Insert (a) CRT cooperates with CNX and PDIA3 to form the CNX/CRT cycle that controls protein folding within the ER. The CNX/CRT cycle can specifically recognize glycoproteins linked by N2 glycosidic bonds and regulate Ca^2+^ homeostasis and Ca^2+^ signaling processes in the ER. Insert (b) Bip is involved in a variety of ER functions dependent on ATP-induced conformational changes. The adenosine triphosphatase (ATPase) cycle of Bip is regulated by its co-chaperones of two families, namely, the ER-localized DnaJ-like proteins (ERdjs) and the nucleotide exchange factors (NEFs). ERdjs enables Bip to stably associate with its target protein. The NEF SIL1 then mediates the release of the target protein from the upper Bip and further folds to form the mature protein. Modified from Fucikova et al. (2021), Ichhaporia and Hendershot (2021).

A considerable amount of evidence indicates that ER chaperones play key roles in neural development through various processes, such as neuronal migration and localization, neuronal morphogenesis, and synaptic modification. There are two main mechanisms by which ER chaperones participate in the regulation of neural development: 1. ER chaperones directly act on specific developmental periods to regulate developmental stages. 2. ER chaperones indirectly regulate development by regulating the spatial and temporal expression of secreted proteins or central development-related receptor proteins. Of these methods, the indirect regulation mode mainly affects the secretory protein Reelin and membrane receptor proteins AMPA receptor, NMDA receptor and Wnt receptor. Bip protein maintains protein homeostasis under ER stress. In addition, the key effects of these partners in neurodevelopmental diseases also deserve our attention (Table 1). Bip, SIL1, and PDI-1 regulate neuronal migration. Bip, CNX, CRT and PFDN regulate early embryonic development. CNX, CRT and Sig-1R regulate neuronal morphogenesis and dendritic axon growth. Bip, CNX, and Sig-1R regulate synaptic function (Figure 1). These direct effects occur through membrane receptor-independent mechanism and are related to the regulation of ER calcium levels by ER chaperones. However, the direct mechanisms of action remain unclear and need to be further studied.

**TABLE 1:** The role of Endoplasmic reticulum (ER) chaperones in neural development.

Protein	Localization	Development stage	Neurodevelopmental disorders	References
GRP78/Bip	ER lumen ER *trans*-membrane Cell surface Nucleus	Embryonic neurodevelopment Neuronal migration Synapse formation and plasticity	BD	Chen et al., 2000; Abood et al., 2001; Bown et al., 2002; Nadarajah and Parnavelas, 2002; Kim et al., 2005; Aghajani et al., 2006; Luo et al., 2006; So et al., 2007; Mimura et al., 2008; Zhang and Kaufman, 2008; Hayashi et al., 2009; Rossi et al., 2010; van Schadewijk et al., 2012; Chiarlone et al., 2014; Jin et al., 2014, 2017; Pfaffenseller et al., 2014; Wang J. et al., 2017; Bengesser et al., 2018; Gómez-Almería et al., 2021
SIL1	ER lumen	Neuronal migration	ID	Krieger et al., 2013; Inaguma et al., 2014; Roos et al., 2014; Osborn et al., 2017; Wang J. et al., 2017
Calnexin	ER *trans*-membrane Cell surface	Embryonic neurodevelopment Axon/dendrite initiation Synapse formation and plasticity	ID	Wraith et al., 1987; Wilson et al., 1990; Tjoelker et al., 1994; Gloor et al., 2001; Denzel et al., 2002; Coe et al., 2008; Smith et al., 2008; Kraus et al., 2010; Itakura et al., 2013; Ficicioglu et al., 2018; Jung et al., 2018; Stapleton et al., 2018; Osaki et al., 2019
Calreticulin	ER lumen Cytosol Cell surface	Embryonic neurodevelopment Axon/dendrite initiation Neuronal differentiation	ASD	Solheim et al., 1997; Mesaeli et al., 1999; Oldenborg et al., 2000; Rauch et al., 2000; Bartholomeusz et al., 2002; Chao, 2003; Willis et al., 2005; Kumar et al., 2008; Weiss et al., 2008; McCarthy et al., 2009; Vuppalanchi et al., 2010; Hazlett et al., 2011; Shih et al., 2012; Maillard et al., 2015; Sacco et al., 2015; Lee et al., 2019; Zarrei et al., 2019; Pacheco et al., 2020; Pilquil et al., 2020; Li et al., 2021
PDI	ER lumen Cell surface	Neuronal migration	ASD, ID	Desai et al., 1988; Whangbo and Kenyon, 1999; Turano et al., 2002; Wilkinson and Gilbert, 2004; Ellgaard and Ruddock, 2005; Fatemi et al., 2005; Iossifov et al., 2014; Wang et al., 2014; Parakh and Atkin, 2015; Lammert and Howell, 2016; Perri et al., 2016; Zeeshan et al., 2016; Lammert et al., 2017; Martin et al., 2018; Torpe et al., 2019; Bilches Medinas et al., 2022
Sigma-1 Receptor	ER *trans*-membrane	Axon/dendrite initiation Spine maturation Synapse formation and plasticity	ASD, ID, ADHD	Barkley et al., 1992; Gibbons et al., 1992, 1995, 2008; Bakker et al., 1994; Matsuno et al., 1996; Amir et al., 1999; Kaufmann and Moser, 2000; Matsumoto et al., 2001; Aydar et al., 2002; Maurice et al., 2002; Amini et al., 2004; Hayashi and Su, 2004; Rizzuto et al., 2004; Arnsten and Dudley, 2005; Arnsten and Li, 2005; Capp et al., 2005; Arnsten, 2006; Csordás et al., 2006; Laurvick et al., 2006; Mientjes et al., 2006; Szabadkai et al., 2006; Kann and Kovács, 2007; Fontanilla et al., 2009; Hagerman et al., 2009; Tsai et al., 2009; Zheng, 2009; Boyle and Kaufmann, 2010; Cárdenas et al., 2010; Neul et al., 2010, 2014; Su et al., 2010, 2016; Budimirovic and Kaufmann, 2011; Nogami et al., 2011; Ruscher et al., 2011, 2012; Shioda et al., 2011; Villard et al., 2011; Ishima and Hashimoto, 2012; Kourrich et al., 2012; Levenga and Willemsen, 2012; Zhang C. L. et al., 2012; Mori et al., 2013; Samaco et al., 2013; Ishima et al., 2014; Peviani et al., 2014; Robson et al., 2014; Lombardi et al., 2015; Kaufmann et al., 2016, 2017, 2019; Xavier et al., 2016; Fukunaga and Moriguchi, 2017; Dahlhaus, 2018; Yamaguchi et al., 2018; Ryskamp et al., 2019; Schmidt and Kruse, 2019; Tan and Zoghbi, 2019; Hampel et al., 2020; Motawe et al., 2020; Reyes et al., 2021; Dalwadi et al., 2022
Prefoldin	ER lumen Nucleus Cytosol	Embryonic neurodevelopment	N.D.	Cao et al., 2008; Monzo et al., 2010; Lee et al., 2011; Delgehyr et al., 2012; Glover and Clark, 2015; Bertling and Hotulainen, 2017

In summary, considerable research on the function of ER molecular chaperones has been performed, and some progress has been made. However, some limitations remain. 1. ER molecular chaperones have specific expression sites in the early stage of neural development, and specific spatial and temporal patterns coincide with different stages of neural development. For example, Bip is involved in early embryonic development and is closely related to cortical neuronal migration. Sig-1R regulates neuronal morphogenesis and dendritic axon growth and are mainly localized in the hippocampus. Do these findings suggest that the functions of these molecular chaperones are focused at specific developmental sites at specific developmental times? 2. The mechanism by which ER chaperones affect central nervous system development is still not well defined. For example, Bip and SIL1 coregulate the coordination mechanism during central development. CNX regulates the specific regulatory processes of the NMDA receptor and AMPA receptor. Whether PDI-mediated Wnt protein-dependent disulfide bond formation affects other developmental processes in addition to migration remains unclear. As well characterized molecular ER chaperone, the role of GRP94 in protein folding and assembly as well as in cancer and related diseases has been widely studied (Fu and Lee, 2006; Pan et al., 2009). However, there are few studies on GRP94 in neural development, and further exploration is needed. 3. At present, studies on the role of molecular chaperones in developmental function are insufficient, and further studies on the functions of these molecular chaperones in different brain regions are needed. Finally, studies on the key role and induction mechanism of molecular chaperones in neurodevelopmental diseases are limited.

At present, neurodevelopmental diseases have become a problem of great concern. The complex etiology and specific pathogenesis have affected the health of numerous individuals. Determining whether it is possible to repair developmental defects by controlling the expression of chaperone proteins and subsequently treat developmental diseases is the focus of our research.

## Author contributions

XZ and JZ: conceptualization and funding acquisition. HS, MWu, MWa, and XZ: writing—original draft preparation. HS, MWu, and XZ: writing—review and editing. All authors contributed to the article and approved the submitted version.

## References

[B1] Abood M. E. Rizvi G. Sallapudi N. McAllister S. D. (2001). Activation of the CB1 cannabinoid receptor protects cultured mouse spinal neurons against excitotoxicity. *Neurosci. Lett.* 309 197–201. 10.1016/s0304-3940(01)02065-1 11514075

[B2] Aghajani A. Rahimi A. Fadai F. Ebrahimi A. Najmabadi H. Ohadi M. (2006). A point mutation at the calreticulin gene core promoter conserved sequence in a case of schizophrenia. *Am. J. Med. Genet. B Neuropsychiatr. Genet.* 141B 294–295. 10.1002/ajmg.b.30300 16511840

[B3] Amini B. Yang P. B. Swann A. C. Dafny N. (2004). Differential locomotor responses in male rats from three strains to acute methylphenidate. *Int. J. Neurosci.* 114 1063–1084. 10.1080/00207450490475526 15370174

[B4] Amir R. E. Van den Veyver I. B. Wan M. Tran C. Q. Francke U. Zoghbi H. Y. (1999). Rett syndrome is caused by mutations in X-linked MECP2, encoding methyl-CpG-binding protein 2. *Nat. Genet.* 23 185–188. 10.1038/13810 10508514

[B5] Apolloni S. Caputi F. Pignataro A. Amadio S. Fabbrizio P. Ammassari-Teule M. (2019). Histamine is an inducer of the heat shock response in SOD1-G93A models of ALS. *Int. J. Mol. Sci.* 20:3793. 10.3390/ijms20153793 31382568 PMC6696457

[B6] Arim R. G. Kohen D. E. Garner R. E. Lach L. M. Brehaut J. C. MacKenzie M. J. (2015). Psychosocial functioning in children with neurodevelopmental disorders and externalizing behavior problems. *Disabil. Rehabil.* 37 345–354. 10.3109/09638288.2014.919361 24840026

[B7] Arnsten A. F. (2006). Stimulants: Therapeutic actions in ADHD. *Neuropsychopharmacology* 31 2376–2383. 10.1038/sj.npp.1301164 16855530

[B8] Arnsten A. F. Dudley A. G. (2005). Methylphenidate improves prefrontal cortical cognitive function through alpha2 adrenoceptor and dopamine D1 receptor actions: Relevance to therapeutic effects in attention deficit hyperactivity disorder. *Behav. Brain Funct.* 1:2. 10.1186/1744-9081-1-2 15916700 PMC1143775

[B9] Arnsten A. F. Li B. M. (2005). Neurobiology of executive functions: Catecholamine influences on prefrontal cortical functions. *Biol. Psychiatry* 57 1377–1384. 10.1016/j.biopsych.2004.08.019 15950011

[B10] Aydar E. Palmer C. P. Klyachko V. A. Jackson M. B. (2002). The sigma receptor as a ligand-regulated auxiliary potassium channel subunit. *Neuron* 34 399–410. 10.1016/s0896-6273(02)00677-3 11988171

[B11] Bădescu G. M. Fîlfan M. Sandu R. E. Surugiu R. Ciobanu O. Popa-Wagner A. (2016). Molecular mechanisms underlying neurodevelopmental disorders, ADHD and autism. *Rom. J. Morphol. Embryol.* 57 361–366. 27516006

[B12] Bagni C. Zukin R. S. (2019). A synaptic perspective of fragile X syndrome and autism spectrum disorders. *Neuron* 101 1070–1088. 10.1016/j.neuron.2019.02.041 30897358 PMC9628679

[B13] Bakker C. E. Verheij C. Willemsen R. van der Helm R. Oerlemans F. Vermey M. (1994). Fmr1 knockout mice: A model to study fragile X mental retardation. The dutch-belgian fragile X consortium. *Cell* 78 23–33. 8033209

[B14] Barkley R. A. Grodzinsky G. DuPaul G. J. (1992). Frontal lobe functions in attention deficit disorder with and without hyperactivity: A review and research report. *J. Abnorm. Child Psychol.* 20 163–188. 10.1007/BF00916547 1593025

[B15] Bartholomeusz H. H. Courchesne E. Karns C. M. (2002). Relationship between head circumference and brain volume in healthy normal toddlers, children, and adults. *Neuropediatrics* 33 239–241. 10.1055/s-2002-36735 12536365

[B16] Baxter A. J. Brugha T. S. Erskine H. E. Scheurer R. W. Vos T. Scott J. G. (2015). The epidemiology and global burden of autism spectrum disorders. *Psychol. Med.* 45 601–613. 10.1017/S003329171400172X 25108395

[B17] Beghi E. Chiò A. Couratier P. Esteban J. Hardiman O. Logroscino G. (2011). The epidemiology and treatment of ALS: Focus on the heterogeneity of the disease and critical appraisal of therapeutic trials. *Amyotroph. Lateral Scler.* 12 1–10. 10.3109/17482968.2010.502940 20698807 PMC3513399

[B18] Belmonte-Mateos C. Pujades C. (2022). From cell states to cell fates: How cell proliferation and neuronal differentiation are coordinated during embryonic development. *Front. Neurosci.* 15:781160. 10.3389/fnins.2021.781160 35046768 PMC8761814

[B19] Bengesser S. A. Reininghaus E. Z. Dalkner N. Birner A. Hohenberger H. Queissner R. (2018). Endoplasmic reticulum stress in bipolar disorder? – BiP and CHOP gene expression- and XBP1 splicing analysis in peripheral blood. *Psychoneuroendocrinology* 95 113–119. 10.1016/j.psyneuen.2018.05.029 29843019

[B20] Bertling E. Hotulainen P. (2017). New waves in dendritic spine actin cytoskeleton: From branches and bundles to rings, from actin binding proteins to post-translational modifications. *Mol. Cell. Neurosci.* 84 77–84. 10.1016/j.mcn.2017.05.002 28479292

[B21] Bilches Medinas D. Malik S. Yıldız-Bölükbaşı E. Borgonovo J. Saaranen M. J. Urra H. (2022). Mutation in protein disulfide isomerase A3 causes neurodevelopmental defects by disturbing endoplasmic reticulum proteostasis. *EMBO J.* 41:e105531. 10.15252/embj.2020105531 34904718 PMC8762563

[B22] Bocchi R. Egervari K. Carol-Perdiguer L. Viale B. Quairiaux C. De Roo M. (2017). Perturbed Wnt signaling leads to neuronal migration delay, altered interhemispheric connections and impaired social behavior. *Nat. Commun.* 8:1158. 10.1038/s41467-017-01046-w 29079819 PMC5660087

[B23] Bose D. Chakrabarti A. (2017). Substrate specificity in the context of molecular chaperones. *IUBMB Life* 69 647–659. 10.1002/iub.1656 28748601

[B24] Bostan A. C. Dum R. P. Strick P. L. (2018). Functional anatomy of basal ganglia circuits with the cerebral cortex and the cerebellum. *Prog. Neurol. Surg.* 33 50–61. 10.1159/000480748 29332073

[B25] Bown C. D. Wang J. F. Chen B. Young L. T. (2002). Regulation of ER stress proteins by valproate: Therapeutic implications. *Bipolar Disord.* 4 145–151. 10.1034/j.1399-5618.2002.t01-1-40201.x 12071512

[B26] Boyle L. Kaufmann W. E. (2010). The behavioral phenotype of FMR1 mutations. *Am. J. Med. Genet. C Semin. Med. Genet.* 154C 469–476. 10.1002/ajmg.c.30277 20981777

[B27] Bradke F. Fawcett J. W. Spira M. E. (2012). Assembly of a new growth cone after axotomy: The precursor to axon regeneration. *Nat. Rev. Neurosci.* 13 183–193. 10.1038/nrn3176 22334213

[B28] Bramham C. R. Alme M. N. Bittins M. Kuipers S. D. Nair R. R. Pai B. (2010). The Arc of synaptic memory. *Exp. Brain Res.* 200 125–140. 10.1007/s00221-009-1959-2 19690847 PMC2803749

[B29] Budimirovic D. B. Kaufmann W. E. (2011). What can we learn about autism from studying fragile X syndrome? *Dev. Neurosci.* 33 379–394. 10.1159/000330213 21893949 PMC3254037

[B30] Buffington S. A. Huang W. Costa-Mattioli M. (2014). Translational control in synaptic plasticity and cognitive dysfunction. *Annu. Rev. Neurosci.* 37 17–38. 10.1146/annurev-neuro-071013-014100 25032491 PMC4721605

[B31] Cao S. Carlesso G. Osipovich A. B. Llanes J. Lin Q. Hoek K. L. (2008). Subunit 1 of the prefoldin chaperone complex is required for lymphocyte development and function. *J. Immunol.* 181 476–484. 10.4049/jimmunol.181.1.476 18566413

[B32] Capp P. K. Pearl P. L. Conlon C. (2005). Methylphenidate HCl: Therapy for attention deficit hyperactivity disorder. *Expert Rev. Neurother.* 5 325–331. 10.1586/14737175.5.3.325 15938665

[B33] Cárdenas C. Miller R. A. Smith I. Bui T. Molgó J. Müller M. (2010). Essential regulation of cell bioenergetics by constitutive InsP3 receptor Ca2+ transfer to mitochondria. *Cell* 142 270–283. 10.1016/j.cell.2010.06.007 20655468 PMC2911450

[B34] Carver J. A. Ecroyd H. Truscott R. Thorn D. C. Holt C. (2018). Proteostasis and the regulation of intra- and extracellular protein aggregation by ATP-independent molecular chaperones: Lens α-crystallins and milk caseins. *Acc. Chem. Res.* 51 745–752. 10.1021/acs.accounts.7b00250 29442498

[B35] Castelo-Branco G. Wagner J. Rodriguez F. J. Kele J. Sousa K. Rawal N. (2003). Differential regulation of midbrain dopaminergic neuron development by Wnt-1, Wnt-3a, and Wnt-5a. *Proc. Natl. Acad. Sci. U.S.A.* 100 12747–12752. 10.1073/pnas.1534900100 14557550 PMC240689

[B36] Caviness V. S. Jr. (1982). Neocortical histogenesis in normal and reeler mice: A developmental study based upon [3H]thymidine autoradiography. *Brain Res.* 256 293–302. 10.1016/0165-3806(82)90141-9 7104762

[B37] Chao M. V. (2003). Neurotrophins and their receptors: A convergence point for many signalling pathways. *Nat. Rev. Neurosci.* 4 299–309. 10.1038/nrn1078 12671646

[B38] Chen B. Wang J. F. Young L. T. (2000). Chronic valproate treatment increases expression of endoplasmic reticulum stress proteins in the rat cerebral cortex and hippocampus. *Biol. Psychiatry* 48 658–664. 10.1016/s0006-3223(00)00878-7 11032977

[B39] Chen B. Wu Z. Xu J. Xu Y. (2015). Calreticulin binds to fas ligand and inhibits neuronal cell apoptosis induced by ischemia-reperfusion injury. *Biomed Res. Int.* 2015:895284. 10.1155/2015/895284 26583143 PMC4637069

[B40] Chiarlone A. Bellocchio L. Blázquez C. Resel E. Soria-Gómez E. Cannich A. (2014). A restricted population of CB1 cannabinoid receptors with neuroprotective activity. *Proc. Natl. Acad. Sci. U.S.A.* 111 8257–8262. 10.1073/pnas.1400988111 24843137 PMC4050577

[B41] Coe H. Bedard K. Groenendyk J. Jung J. Michalak M. (2008). Endoplasmic reticulum stress in the absence of calnexin. *Cell Stress Chaperones* 13 497–507. 10.1007/s12192-008-0049-x 18528784 PMC2673926

[B42] Correll R. N. Grimes K. M. Prasad V. Lynch J. M. Khalil H. Molkentin J. D. (2019). Overlapping and differential functions of ATF6α versus ATF6β in the mouse heart. *Sci. Rep.* 9:2059. 10.1038/s41598-019-39515-5 30765833 PMC6375966

[B43] Coudreuse D. Y. Roël G. Betist M. C. Destrée O. Korswagen H. C. (2006). Wnt gradient formation requires retromer function in Wnt-producing cells. *Science* 312 921–924. 10.1126/science.1124856 16645052

[B44] Crelin E. S. (1974). Development of the nervous system. A logical approach to neuroanatomy. *Clin. Symposia* 26 1–32. 4851346

[B45] Csordás G. Renken C. Várnai P. Walter L. Weaver D. Buttle K. F. (2006). Structural and functional features and significance of the physical linkage between ER and mitochondria. *J. Cell Biol.* 174 915–921. 10.1083/jcb.200604016 16982799 PMC2064383

[B46] Cull-Candy S. Brickley S. Farrant M. (2001). NMDA receptor subunits: Diversity, development and disease. *Curr. Opin. Neurobiol.* 11 327–335. 10.1016/s0959-4388(00)00215-4 11399431

[B47] Dahlhaus R. (2018). Of men and mice: Modeling the fragile X syndrome. *Front. Mol. Neurosci.* 11:41. 10.3389/fnmol.2018.00041 29599705 PMC5862809

[B48] Dalwadi D. A. Kim S. Schetz J. Schreihofer D. A. Kim S. (2022). Brain-derived neurotrophic factor for high-throughput evaluation of selective sigma-1 receptor ligands. *J. Pharmacol. Toxicol. Methods* 113:107129. 10.1016/j.vascn.2021.107129 34678430 PMC9358981

[B49] DaSilva L. L. Wall M. J. P de Almeida L. Wauters S. C. Januário Y. C. Müller J. (2016). Activity-regulated cytoskeleton-associated protein controls AMPAR endocytosis through a direct interaction with clathrin-adaptor protein 2. *eNeuro* 3 1–22. 10.1523/ENEURO.0144-15.2016 27257628 PMC4877669

[B50] de Montigny C. Monnet F. P. Fournier A. Debonnel G. (1992). Sigma ligands and neuropeptide Y selectively potentiate the NMDA response in the rat CA3 dorsal hippocampus: In vivo electrophysiological studies. *Clin. Neuropharmacol.* 15(Suppl. 1) 145A–146A. 10.1097/00002826-199201001-00078 1323387

[B51] Delgehyr N. Wieland U. Rangone H. Pinson X. Mao G. Dzhindzhev N. S. (2012). *Drosophila* Mgr, a Prefoldin subunit cooperating with von Hippel Lindau to regulate tubulin stability. *Proc. Natl. Acad. Sci. U.S.A.* 109 5729–5734. 10.1073/pnas.1108537109 22451918 PMC3326472

[B52] Denzel A. Molinari M. Trigueros C. Martin J. E. Velmurgan S. Brown S. (2002). Early postnatal death and motor disorders in mice congenitally deficient in calnexin expression. *Mol. Cell. Biol.* 22 7398–7404. 10.1128/MCB.22.21.7398-7404.2002 12370287 PMC135653

[B53] Desai C. Garriga G. McIntire S. L. Horvitz H. R. (1988). A genetic pathway for the development of the *Caenorhabditis elegans* HSN motor neurons. *Nature* 336 638–646. 10.1038/336638a0 3200316

[B54] Devakumar D. Bamford A. Ferreira M. U. Broad J. Rosch R. E. Groce N. (2018). Infectious causes of microcephaly: Epidemiology, pathogenesis, diagnosis, and management. *Lancet Infect. Dis.* 18 e1–e13. 10.1016/S1473-3099(17)30398-5 28844634

[B55] Doyle S. M. Genest O. Wickner S. (2013). Protein rescue from aggregates by powerful molecular chaperone machines. *Nat. Rev. Mol. Cell Biol.* 14 617–629. 10.1038/nrm3660 24061228

[B56] Ehlers M. D. (2000). Reinsertion or degradation of AMPA receptors determined by activity-dependent endocytic sorting. *Neuron* 28 511–525. 10.1016/s0896-6273(00)00129-x 11144360

[B57] Ellgaard L. Ruddock L. W. (2005). The human protein disulphide isomerase family: Substrate interactions and functional properties. *EMBO Rep.* 6 28–32. 10.1038/sj.embor.7400311 15643448 PMC1299221

[B58] Eyre O. Hughes R. A. Thapar A. K. Leibenluft E. Stringaris A. Davey Smith G. (2019). Childhood neurodevelopmental difficulties and risk of adolescent depression: The role of irritability. *J. Child Psychol. Psychiatry* 60 866–874. 10.1111/jcpp.13053 30908655 PMC6767365

[B59] Fatemi S. H. Snow A. V. Stary J. M. Araghi-Niknam M. Reutiman T. J. Lee S. (2005). Reelin signaling is impaired in autism. *Biol. Psychiatry* 57 777–787. 15820235 10.1016/j.biopsych.2004.12.018

[B60] Fernández-Fernández M. R. Valpuesta J. M. (2018). Hsp70 chaperone: A master player in protein homeostasis. *F1000Res.* 7 1–10. 10.12688/f1000research.15528.1 30338057 PMC6148205

[B61] Ficicioglu C. Giugliani R. Harmatz P. Mendelsohn N. J. Jego V. Parini R. (2018). Intrafamilial variability in the clinical manifestations of mucopolysaccharidosis type II: Data from the hunter outcome survey (HOS). *Am. J. Med. Genet. A* 176 301–310. 10.1002/ajmg.a.38551 29210515 PMC5814921

[B62] Filareti M. Luotti S. Pasetto L. Pignataro M. Paolella K. Messina P. (2017). Decreased levels of foldase and chaperone proteins are associated with an early-onset amyotrophic lateral sclerosis. *Front. Mol. Neurosci.* 10:99. 10.3389/fnmol.2017.00099 28428745 PMC5382314

[B63] Fontanilla D. Johannessen M. Hajipour A. R. Cozzi N. V. Jackson M. B. Ruoho A. E. (2009). The hallucinogen N,N-dimethyltryptamine (DMT) is an endogenous sigma-1 receptor regulator. *Science* 323 934–937. 10.1126/science.1166127 19213917 PMC2947205

[B64] Freese J. L. Pino D. Pleasure S. J. (2010). Wnt signaling in development and disease. *Neurobiol. Dis.* 38 148–153. 10.1016/j.nbd.2009.09.003 19765659 PMC2854277

[B65] Freilich R. Arhar T. Abrams J. L. Gestwicki J. E. (2018). Protein-protein interactions in the molecular chaperone network. *Acc. Chem. Res.* 51 940–949. 10.1021/acs.accounts.8b00036 29613769 PMC6082625

[B66] Fu Y. Lee A. S. (2006). Glucose regulated proteins in cancer progression, drug resistance and immunotherapy. *Cancer Biol. Ther.* 5 741–744. 10.4161/cbt.5.7.2970 16861902

[B67] Fucikova J. Spisek R. Kroemer G. Galluzzi L. (2021). Calreticulin and cancer. *Cell Res.* 31 5–16. 10.1038/s41422-020-0383-9 32733014 PMC7853084

[B68] Fukunaga K. Moriguchi S. (2017). Stimulation of the sigma-1 receptor and the effects on neurogenesis and depressive behaviors in mice. *Adv. Exp. Med. Biol.* 964 201–211. 10.1007/978-3-319-50174-1_14 28315273

[B69] Galligan J. J. Petersen D. R. (2012). The human protein disulfide isomerase gene family. *Hum. Genomics* 6:6. 10.1186/1479-7364-6-6 23245351 PMC3500226

[B70] Gerrow K. Triller A. (2010). Synaptic stability and plasticity in a floating world. *Curr. Opin. Neurobiol.* 20 631–639. 10.1016/j.conb.2010.06.010 20655734

[B71] Gewirth D. T. (2016). Paralog specific Hsp90 inhibitors – a brief history and a bright future. *Curr. Top. Med. Chem.* 16 2779–2791. 10.2174/1568026616666160413141154 27072700 PMC4995118

[B72] Ghashghaei H. T. Lai C. Anton E. S. (2007). Neuronal migration in the adult brain: Are we there yet? *Nat. Rev. Neurosci.* 8 141–151. 10.1038/nrn2074 17237805

[B73] Gibbons R. J. Picketts D. J. Villard L. Higgs D. R. (1995). Mutations in a putative global transcriptional regulator cause X-linked mental retardation with alpha-thalassemia (ATR-X syndrome). *Cell* 80 837–845. 10.1016/0092-8674(95)90287-2 7697714

[B74] Gibbons R. J. Suthers G. K. Wilkie A. O. Buckle V. J. Higgs D. R. (1992). X-linked alpha-thalassemia/mental retardation (ATR-X) syndrome: Localization to Xq12-q21.31 by X inactivation and linkage analysis. *Am. J. Hum. Genet.* 51 1136–1149. 1415255 PMC1682840

[B75] Gibbons R. J. Wada T. Fisher C. A. Malik N. Mitson M. J. Steensma D. P. (2008). Mutations in the chromatin-associated protein ATRX. *Hum. Mutat.* 29 796–802. 10.1002/humu.20734 18409179

[B76] Gloor S. M. Wachtel M. Bolliger M. F. Ishihara H. Landmann R. Frei K. (2001). Molecular and cellular permeability control at the blood-brain barrier. *Brain Res.* 36 258–264. 10.1016/s0165-0173(01)00102-3 11690623

[B77] Glover D. J. Clark D. S. (2015). Oligomeric assembly is required for chaperone activity of the filamentous γ-prefoldin. *FEBS J.* 282 2985–2997. 10.1111/febs.13341 26096656

[B78] Gómez-Almería M. Burgaz S. Costas-Insua C. Rodríguez-Cueto C. Santos-García I. Rodríguez-Crespo I. (2021). BiP heterozigosity aggravates pathological deterioration in experimental amyotrophic lateral sclerosis. *Int. J. Mol. Sci.* 22:12533. 10.3390/ijms222212533 34830414 PMC8621319

[B79] Guerrini R. Dobyns W. B. (2014). Malformations of cortical development: Clinical features and genetic causes. *Lancet Neurol.* 13 710–726. 10.1016/S1474-4422(14)70040-7 24932993 PMC5548104

[B80] Guo C. Ma Y. Y. (2021). Calcium permeable-AMPA receptors and excitotoxicity in neurological disorders. *Front. Neural Circuits* 15:711564. 10.3389/fncir.2021.711564 34483848 PMC8416103

[B81] Hagerman R. J. Berry-Kravis E. Kaufmann W. E. Ono M. Y. Tartaglia N. Lachiewicz A. (2009). Advances in the treatment of fragile X syndrome. *Pediatrics* 123 378–390. 10.1542/peds.2008-0317 19117905 PMC2888470

[B82] Hampel H. Williams C. Etcheto A. Goodsaid F. Parmentier F. Sallantin J. (2020). A precision medicine framework using artificial intelligence for the identification and confirmation of genomic biomarkers of response to an Alzheimer’s disease therapy: Analysis of the blarcamesine (ANAVEX2-73) Phase 2a clinical study. *Alzheimers Dement.* 6:e12013. 10.1002/trc2.12013 32318621 PMC7167374

[B83] Hanner M. Moebius F. F. Flandorfer A. Knaus H. G. Striessnig J. Kempner E. (1996). Purification, molecular cloning, and expression of the mammalian sigma1-binding site. *Proc. Natl. Acad. Sci. U.S.A.* 93 8072–8077. 10.1073/pnas.93.15.8072 8755605 PMC38877

[B84] Hayashi A. Kasahara T. Kametani M. Toyota T. Yoshikawa T. Kato T. (2009). Aberrant endoplasmic reticulum stress response in lymphoblastoid cells from patients with bipolar disorder. *Int. J. Neuropsychopharmacol.* 12 33–43. 10.1017/S1461145708009358 18771604

[B85] Hayashi T. Su T. P. (2004). Sigma-1 receptor ligands: Potential in the treatment of neuropsychiatric disorders. *CNS Drugs* 18 269–284. 10.2165/00023210-200418050-00001 15089113

[B86] Hayashi T. Su T. P. (2007). Sigma-1 receptor chaperones at the ER-mitochondrion interface regulate Ca(2+) signaling and cell survival. *Cell* 131 596–610. 10.1016/j.cell.2007.08.036 17981125

[B87] Hazlett H. C. Poe M. D. Gerig G. Styner M. Chappell C. Smith R. G. (2011). Early brain overgrowth in autism associated with an increase in cortical surface area before age 2 years. *Arch. Gen. Psychiatry* 68 467–476. 10.1001/archgenpsychiatry.2011.39 21536976 PMC3315057

[B88] Helenius A. Aebi M. (2001). Intracellular functions of N-linked glycans. *Science* 291 2364–2369. 10.1126/science.291.5512.2364 11269317

[B89] Hendershot L. Wei J. Gaut J. Melnick J. Aviel S. Argon Y. (1996). Inhibition of immunoglobulin folding and secretion by dominant negative BiP ATPase mutants. *Proc. Natl. Acad. Sci. U.S.A.* 93 5269–5274. 10.1073/pnas.93.11.5269 8643565 PMC39234

[B90] Henley J. M. Wilkinson K. A. (2016). Synaptic AMPA receptor composition in development, plasticity and disease. *Nat. Rev. Neurosci.* 17 337–350. 10.1038/nrn.2016.37 27080385

[B91] Hilliard M. A. Bargmann C. I. (2006). Wnt signals and frizzled activity orient anterior-posterior axon outgrowth in *C. elegans*. *Dev. Cell* 10 379–390. 10.1016/j.devcel.2006.01.013 16516840

[B92] Hinata M. Matsuoka I. Iwamoto T. Watanabe Y. Kimura J. (2007). Mechanism of Na+/Ca2+ exchanger activation by hydrogen peroxide in guinea-pig ventricular myocytes. *J. Pharmacol. Sci.* 103 283–292. 10.1254/jphs.fp0060015 17332693

[B93] Hoter A. El-Sabban M. E. Naim H. Y. (2018). The HSP90 family: Structure, regulation, function, and implications in health and disease. *Int. J. Mol. Sci.* 19:2560. 10.3390/ijms19092560 30158430 PMC6164434

[B94] Ichhaporia V. P. Hendershot L. M. (2021). Role of the HSP70 co-chaperone sil1 in health and disease. *Int. J. Mol. Sci.* 22:1564. 10.3390/ijms22041564 33557244 PMC7913895

[B95] Inaguma Y. Hamada N. Tabata H. Iwamoto I. Mizuno M. Nishimura Y. V. (2014). SIL1, a causative cochaperone gene of marinesco-söjgren syndrome, plays an essential role in establishing the architecture of the developing cerebral cortex. *EMBO Mol. Med.* 6 414–429. 10.1002/emmm.201303069 24473200 PMC3958314

[B96] Iossifov I. O’Roak B. J. Sanders S. J. Ronemus M. Krumm N. Levy D. (2014). The contribution of de novo coding mutations to autism spectrum disorder. *Nature* 515 216–221. 10.1038/nature13908 25363768 PMC4313871

[B97] Ishima T. Hashimoto K. (2012). Potentiation of nerve growth factor-induced neurite outgrowth in PC12 cells by ifenprodil: The role of sigma-1 and IP3 receptors. *PLoS One* 7:e37989. 10.1371/journal.pone.0037989 22655093 PMC3360021

[B98] Ishima T. Fujita Y. Hashimoto K. (2014). Interaction of new antidepressants with sigma-1 receptor chaperones and their potentiation of neurite outgrowth in PC12 cells. *Eur. J. Pharmacol.* 727 167–173. 10.1016/j.ejphar.2014.01.064 24508523

[B99] Ismail F. Y. Shapiro B. K. (2019). What are neurodevelopmental disorders? *Curr. Opin. Neurol.* 32 611–616. 10.1097/WCO.0000000000000710 31116115

[B100] Itakura M. Tsujimura J. Yamamori S. Ohkido T. Takahashi M. (2013). NMDA receptor-dependent recruitment of calnexin to the neuronal plasma membrane. *Neurosci. Lett.* 550 173–178. 10.1016/j.neulet.2013.06.064 23851254

[B101] Jiang Y. S. Xu Z. H. (2019). Brain developmental diseases and pathogenic mechanisms. *Hereditas* 41 801–815. 10.16288/j.yczz.19-133 31549679

[B102] Jin H. Komita M. Aoe T. (2017). The role of BiP retrieval by the KDEL receptor in the early secretory pathway and its effect on protein quality control and neurodegeneration. *Front. Mol. Neurosci.* 10:222. 10.3389/fnmol.2017.00222 28769758 PMC5511815

[B103] Jin H. Mimura N. Kashio M. Koseki H. Aoe T. (2014). Late-onset of spinal neurodegeneration in knock-in mice expressing a mutant BiP. *PLoS One* 9:e112837. 10.1371/journal.pone.0112837 25405877 PMC4236098

[B104] Jossin Y. Goffinet A. M. (2007). Reelin signals through phosphatidylinositol 3-kinase and Akt to control cortical development and through mTor to regulate dendritic growth. *Mol. Cell. Biol.* 27 7113–7124. 10.1128/MCB.00928-07 17698586 PMC2168915

[B105] Jung J. Eggleton P. Robinson A. Wang J. Gutowski N. Holley J. (2018). Calnexin is necessary for T cell transmigration into the central nervous system. *JCI Insight* 3:e98410. 10.1172/jci.insight.98410 29515033 PMC5922283

[B106] Kadoyama K. Matsuura K. Takano M. Maekura K. Inoue Y. Matsuyama S. (2019). Changes in the expression of prefoldin subunit 5 depending on synaptic plasticity in the mouse hippocampus. *Neurosci. Lett.* 712:134484. 10.1016/j.neulet.2019.134484 31505240

[B107] Kann O. Kovács R. (2007). Mitochondria and neuronal activity. *Am. J. Physiol. Cell Physiol.* 292 C641–C657. 10.1152/ajpcell.00222.2006 17092996

[B108] Kaufman L. Ayub M. Vincent J. B. (2010). The genetic basis of non-syndromic intellectual disability: A review. *J. Neurodev. Disord.* 2 182–209. 10.1007/s11689-010-9055-2 21124998 PMC2974911

[B109] Kaufmann W. E. Moser H. W. (2000). Dendritic anomalies in disorders associated with mental retardation. *Cereb. Cortex* 10 981–991. 10.1093/cercor/10.10.981 11007549

[B110] Kaufmann W. E. Kidd S. A. Andrews H. F. Budimirovic D. B. Esler A. Haas-Givler B. (2017). Autism spectrum disorder in fragile X syndrome: Cooccurring conditions and current treatment. *Pediatrics* 139(Suppl. 3) S194–S206. 10.1542/peds.2016-1159F 28814540 PMC5619699

[B111] Kaufmann W. E. Sprouse J. Rebowe N. Hanania T. Klamer D. Missling C. U. (2019). ANAVEX§2-73 (blarcamesine), a sigma-1 receptor agonist, ameliorates neurologic impairments in a mouse model of rett syndrome. *Pharmacol. Biochem. Behav.* 187:172796. 10.1016/j.pbb.2019.172796 31704481

[B112] Kaufmann W. E. Stallworth J. L. Everman D. B. Skinner S. A. (2016). Neurobiologically-based treatments in rett syndrome: Opportunities and challenges. *Expert Opin. Orphan Drugs* 4 1043–1055. 10.1080/21678707.2016.1229181 28163986 PMC5214376

[B113] Kessels H. W. Malinow R. (2009). Synaptic AMPA receptor plasticity and behavior. *Neuron* 61 340–350. 10.1016/j.neuron.2009.01.015 19217372 PMC3917551

[B114] Kim A. J. Shi Y. Austin R. C. Werstuck G. H. (2005). Valproate protects cells from ER stress-induced lipid accumulation and apoptosis by inhibiting glycogen synthase kinase-3. *J. Cell Sci.* 118(Pt 1) 89–99. 10.1242/jcs.01562 15585578

[B115] Kimura K. Jin H. Ogawa M. Aoe T. (2008). Dysfunction of the ER chaperone BiP accelerates the renal tubular injury. *Biochem. Biophys. Res. Commun.* 366 1048–1053. 10.1016/j.bbrc.2007.12.098 18158912

[B116] Korb E. Finkbeiner S. (2011). Arc in synaptic plasticity: From gene to behavior. *Trends Neurosci.* 34 591–598. 10.1016/j.tins.2011.08.007 21963089 PMC3207967

[B117] Kourrich S. Su T. P. Fujimoto M. Bonci A. (2012). The sigma-1 receptor: Roles in neuronal plasticity and disease. *Trends Neurosci.* 35 762–771. 10.1016/j.tins.2012.09.007 23102998 PMC3587126

[B118] Kraus A. Groenendyk J. Bedard K. Baldwin T. A. Krause K. H. Dubois-Dauphin M. (2010). Calnexin deficiency leads to dysmyelination. *J. Biol. Chem.* 285 18928–18938. 10.1074/jbc.M110.107201 20400506 PMC2881815

[B119] Krieger M. Roos A. Stendel C. Claeys K. G. Sonmez F. M. Baudis M. (2013). SIL1 mutations and clinical spectrum in patients with marinesco-sjogren syndrome. *Brain* 136(Pt 12) 3634–3644. 10.1093/brain/awt283 24176978

[B120] Kumar R. A. KaraMohamed S. Sudi J. Conrad D. F. Brune C. Badner J. A. (2008). Recurrent 16p11.2 microdeletions in autism. *Hum. Mol. Genet.* 17 628–638. 10.1093/hmg/ddm376 18156158

[B121] Kwan K. Y. Sestan N. Anton E. S. (2012). Transcriptional co-regulation of neuronal migration and laminar identity in the neocortex. *Development* 139 1535–1546. 10.1242/dev.069963 22492350 PMC3317962

[B122] Kwon O. C. Lee E. J. Lee J. Y. Youn J. Kim T. H. Hong S. (2019). Prefoldin 5 and anti-prefoldin 5 antibodies as biomarkers for uveitis in ankylosing spondylitis. *Front. Immunol.* 10:384. 10.3389/fimmu.2019.00384 30891043 PMC6411661

[B123] Lai M. C. Lombardo M. V. Baron-Cohen S. (2014). Autism. *Lancet* 383 896–910. 10.1016/S0140-6736(13)61539-1 24074734

[B124] Lammert D. B. Howell B. W. (2016). RELN mutations in autism spectrum disorder. *Front. Cell. Neurosci.* 10:84. 10.3389/fncel.2016.00084 27064498 PMC4814460

[B125] Lammert D. B. Middleton F. A. Pan J. Olson E. C. Howell B. W. (2017). The de novo autism spectrum disorder RELN R2290C mutation reduces reelin secretion and increases protein disulfide isomerase expression. *J. Neurochem.* 142 89–102. 10.1111/jnc.14045 28419454 PMC6091860

[B126] Laurvick C. L. de Klerk N. Bower C. Christodoulou J. Ravine D. Ellaway C. (2006). Rett syndrome in Australia: A review of the epidemiology. *J. Pediatr.* 148 347–352. 10.1016/j.jpeds.2005.10.037 16615965

[B127] Lee A. C. Shih Y. Y. Zhou F. Chao T. C. Lee H. Liao Y. F. (2019). Calreticulin regulates MYCN expression to control neuronal differentiation and stemness of neuroblastoma. *J. Mol. Med.* 97 325–339. 10.1007/s00109-018-1730-x 30612140

[B128] Lee Y. Smith R. S. Jordan W. King B. L. Won J. Valpuesta J. M. (2011). Prefoldin 5 is required for normal sensory and neuronal development in a murine model. *J. Biol. Chem.* 286 726–736. 10.1074/jbc.M110.177352 20956523 PMC3013031

[B129] Leemhuis J. Bock H. H. (2011). Reelin modulates cytoskeletal organization by regulating Rho GTPases. *Commun. Integr. Biol.* 4 254–257. 10.4161/cib.4.3.14890 21980553 PMC3187881

[B130] Levenga J. Willemsen R. (2012). Perturbation of dendritic protrusions in intellectual disability. *Prog. Brain Res.* 197 153–168. 10.1016/B978-0-444-54299-1.00008-X 22541292

[B131] Li G. Pleasure S. J. (2014). The development of hippocampal cellular assemblies. *Wiley Interdiscip. Rev. Dev. Biol.* 3 165–177. 10.1002/wdev.127 24719288

[B132] Li H. D. Liu W. X. Michalak M. (2011). Enhanced clathrin-dependent endocytosis in the absence of calnexin. *PLoS One* 6:e21678. 10.1371/journal.pone.0021678 21747946 PMC3128601

[B133] Li J. Brickler T. Banuelos A. Marjon K. Shcherbina A. Banerjee S. (2021). Overexpression of CD47 is associated with brain overgrowth and 16p11.2 deletion syndrome. *Proc. Natl. Acad. Sci. U.S.A.* 118:e2005483118. 10.1073/pnas.2005483118 33833053 PMC8053942

[B134] Lièvremont J. P. Rizzuto R. Hendershot L. Meldolesi J. (1997). BiP, a major chaperone protein of the endoplasmic reticulum lumen, plays a direct and important role in the storage of the rapidly exchanging pool of Ca2+. *J. Biol. Chem.* 272 30873–30879. 10.1074/jbc.272.49.30873 9388233

[B135] Liu X. B. Murray K. D. Jones E. G. (2004). Switching of NMDA receptor 2A and 2B subunits at thalamic and cortical synapses during early postnatal development. *J. Neurosci.* 24 8885–8895. 10.1523/JNEUROSCI.2476-04.2004 15470155 PMC6729956

[B136] Lombardi L. M. Baker S. A. Zoghbi H. Y. (2015). MECP2 disorders: From the clinic to mice and back. *J. Clin. Investig.* 125 2914–2923. 10.1172/JCI78167 26237041 PMC4563741

[B137] Lopez de Armentia M. Sah P. (2003). Development and subunit composition of synaptic NMDA receptors in the amygdala: NR2B synapses in the adult central amygdala. *J. Neurosci.* 23 6876–6883. 10.1523/JNEUROSCI.23-17-06876.2003 12890782 PMC6740716

[B138] Luo S. Mao C. Lee B. Lee A. S. (2006). GRP78/BiP is required for cell proliferation and protecting the inner cell mass from apoptosis during early mouse embryonic development. *Mol. Cell. Biol.* 26 5688–5697. 10.1128/MCB.00779-06 16847323 PMC1592753

[B139] Lynch J. M. Maillet M. Vanhoutte D. Schloemer A. Sargent M. A. Blair N. S. (2012). A thrombospondin-dependent pathway for a protective ER stress response. *Cell* 149 1257–1268. 10.1016/j.cell.2012.03.050 22682248 PMC3372931

[B140] Ma F. H. Li C. Liu Y. Shi L. (2020). Mimicking molecular chaperones to regulate protein folding. *Adv. Mater.* 32:e1805945. 10.1002/adma.201805945 31045287

[B141] MacDonald B. T. Hien A. Zhang X. Iranloye O. Virshup D. M. Waterman M. L. (2014). Disulfide bond requirements for active Wnt ligands. *J. Biol. Chem.* 289 18122–18136. 10.1074/jbc.M114.575027 24841207 PMC4140276

[B142] Maillard A. M. Ruef A. Pizzagalli F. Migliavacca E. Hippolyte L. Adaszewski S. (2015). The 16p11.2 locus modulates brain structures common to autism, schizophrenia and obesity. *Mol. Psychiatry* 20 140–147. 10.1038/mp.2014.145 25421402 PMC4320286

[B143] Marín O. Valiente M. Ge X. Tsai L. H. (2010). Guiding neuronal cell migrations. *Cold Spring Harb. Perspect. Biol.* 2:a001834. 10.1101/cshperspect.a001834 20182622 PMC2828271

[B144] Martin H. C. Jones W. D. McIntyre R. Sanchez-Andrade G. Sanderson M. Stephenson J. D. (2018). Quantifying the contribution of recessive coding variation to developmental disorders. *Science* 362 1161–1164. 10.1126/science.aar6731 30409806 PMC6726470

[B145] Matsumoto R. R. Bowen W. D. Tom M. A. Vo V. N. Truong D. D. De Costa B. R. (1995). Characterization of two novel sigma receptor ligands: Antidystonic effects in rats suggest sigma receptor antagonism. *Eur. J. Pharmacol.* 280 301–310. 10.1016/0014-2999(95)00208-3 8566098

[B146] Matsumoto R. R. Hewett K. L. Pouw B. Bowen W. D. Husbands S. M. Cao J. J. (2001). Rimcazole analogs attenuate the convulsive effects of cocaine: Correlation with binding to sigma receptors rather than dopamine transporters. *Neuropharmacology* 41 878–886. 10.1016/s0028-3908(01)00116-2 11684152

[B147] Matsuno K. Nakazawa M. Okamoto K. Kawashima Y. Mita S. (1996). Binding properties of SA4503, a novel and selective sigma 1 receptor agonist. *Eur. J. Pharmacol.* 306 271–279. 10.1016/0014-2999(96)00201-4 8813641

[B148] Maurice T. Martin-Fardon R. Romieu P. Matsumoto R. R. (2002). Sigma(1) (sigma(1)) receptor antagonists represent a new strategy against cocaine addiction and toxicity. *Neurosci. Biobehav. Rev.* 26 499–527. 10.1016/s0149-7634(02)00017-9 12204195

[B149] Mayer M. P. Gierasch L. M. (2019). Recent advances in the structural and mechanistic aspects of Hsp70 molecular chaperones. *J. Biol. Chem.* 294 2085–2097. 10.1074/jbc.REV118.002810 30455352 PMC6369304

[B150] McCarthy S. E. Makarov V. Kirov G. Addington A. M. McClellan J. Yoon S. (2009). Microduplications of 16p11.2 are associated with schizophrenia. *Nat. Genet.* 41 1223–1227. 10.1038/ng.474 19855392 PMC2951180

[B151] McCracken K. A. Bowen W. D. de Costa B. R. Matsumoto R. R. (1999). Two novel sigma receptor ligands, BD1047 and LR172, attenuate cocaine-induced toxicity and locomotor activity. *Eur. J. Pharmacol.* 370 225–232. 10.1016/s0014-2999(99)00113-2 10334496

[B152] Mehregan H. Najmabadi H. Kahrizi K. (2016). Genetic studies in intellectual disability and behavioral impairment. *Arch. Iran. Med.* 19 363–375. 27179170

[B153] Merikangas K. R. Jin R. He J. P. Kessler R. C. Lee S. Sampson N. A. (2011). Prevalence and correlates of bipolar spectrum disorder in the world mental health survey initiative. *Arch. Gen. Psychiatry* 68 241–251. 10.1001/archgenpsychiatry.2011.12 21383262 PMC3486639

[B154] Merlos M. Romero L. Zamanillo D. Plata-Salamán C. Vela J. M. (2017). Sigma-1 receptor and pain. *Handb. Exp. Pharmacol.* 244 131–161. 10.1007/164_2017_9 28275913

[B155] Mesaeli N. Nakamura K. Zvaritch E. Dickie P. Dziak E. Krause K. H. (1999). Calreticulin is essential for cardiac development. *J. Cell Biol.* 144 857–868. 10.1083/jcb.144.5.857 10085286 PMC2148186

[B156] Michalak M. Corbett E. F. Mesaeli N. Nakamura K. Opas M. (1999). Calreticulin: One protein, one gene, many functions. *Biochem. J.* 344(Pt 2) 281–292. 10567207 PMC1220642

[B157] Michalak M. Milner R. E. Burns K. Opas M. (1992). Calreticulin. *Biochem. J.* 285(Pt 3) 681–692. 10.1042/bj2850681 1497605 PMC1132847

[B158] Mientjes E. J. Nieuwenhuizen I. Kirkpatrick L. Zu T. Hoogeveen-Westerveld M. Severijnen L. (2006). The generation of a conditional Fmr1 knock out mouse model to study Fmrp function in vivo. *Neurobiol. Dis.* 21 549–555. 10.1016/j.nbd.2005.08.019 16257225

[B159] Miller D. J. Fort P. E. (2018). Heat shock proteins regulatory role in neurodevelopment. *Front. Neurosci.* 12:821. 10.3389/fnins.2018.00821 30483047 PMC6244093

[B160] Mimura N. Yuasa S. Soma M. Jin H. Kimura K. Goto S. (2008). Altered quality control in the endoplasmic reticulum causes cortical dysplasia in knock-in mice expressing a mutant BiP. *Mol. Cell. Biol.* 28 293–301. 10.1128/MCB.00473-07 17954555 PMC2223281

[B161] Monnet F. P. de Costa B. R. Bowen W. D. (1996). Differentiation of sigma ligand-activated receptor subtypes that modulate NMDA-evoked [3H]-noradrenaline release in rat hippocampal slices. *Br. J. Pharmacol.* 119 65–72. 10.1111/j.1476-5381.1996.tb15678.x 8872358 PMC1915737

[B162] Monnet F. P. Debonnel G. Bergeron R. Gronier B. de Montigny C. (1994). The effects of sigma ligands and of neuropeptide Y on N-methyl-D-aspartate-induced neuronal activation of CA3 dorsal hippocampus neurons are differentially affected by pertussin toxin. *Br. J. Pharmacol.* 112 709–715. 10.1111/j.1476-5381.1994.tb13134.x 8075892 PMC1910385

[B163] Monzo K. Dowd S. R. Minden J. S. Sisson J. C. (2010). Proteomic analysis reveals CCT is a target of Fragile X mental retardation protein regulation in *Drosophila*. *Dev. Biol.* 340 408–418. 10.1016/j.ydbio.2010.01.028 20122915 PMC2857770

[B164] Moon L. D. Xiong F. (2022). Mechanics of neural tube morphogenesis. *Semin. Cell Dev. Biol.* 130 56–69. 10.1016/j.semcdb.2021.09.009 34561169

[B165] Mori T. Hayashi T. Hayashi E. Su T. P. (2013). Sigma-1 receptor chaperone at the ER-mitochondrion interface mediates the mitochondrion-ER-nucleus signaling for cellular survival. *PLoS One* 8:e76941. 10.1371/journal.pone.0076941 24204710 PMC3799859

[B166] Morris-Rosendahl D. J. Crocq M. A. (2020). Neurodevelopmental disorders-the history and future of a diagnostic concept. *Dialogues Clin. Neurosci.* 22 65–72. 10.31887/DCNS.2020.22.1/macrocq 32699506 PMC7365295

[B167] Motawe Z. Y. Abdelmaboud S. S. Cuevas J. Breslin J. W. (2020). PRE-084 as a tool to uncover potential therapeutic applications for selective sigma-1 receptor activation. *Int. J. Biochem. Cell Biol.* 126:105803. 10.1016/j.biocel.2020.105803 32668330 PMC7484451

[B168] Munji R. N. Choe Y. Li G. Siegenthaler J. A. Pleasure S. J. (2011). Wnt signaling regulates neuronal differentiation of cortical intermediate progenitors. *J. Neurosci.* 31 1676–1687. 10.1523/JNEUROSCI.5404-10.2011 21289176 PMC3040956

[B169] Myrum C. Soulé J. Bittins M. Cavagnini K. Goff K. Ziemek S. K. (2017). Arc Interacts with the integral endoplasmic reticulum protein, calnexin. *Front. Cell. Neurosci.* 11:294. 10.3389/fncel.2017.00294 28979192 PMC5611444

[B170] Nadarajah B. Parnavelas J. G. (2002). Modes of neuronal migration in the developing cerebral cortex. *Nat. Rev. Neurosci.* 3 423–432. 10.1038/nrn845 12042877

[B171] Nase G. Weishaupt J. Stern P. Singer W. Monyer H. (1999). Genetic and epigenetic regulation of NMDA receptor expression in the rat visual cortex. *Eur. J. Neurosci.* 11 4320–4326. 10.1046/j.1460-9568.1999.00859.x 10594657

[B172] Neul J. L. Kaufmann W. E. Glaze D. G. Christodoulou J. Clarke A. J. Bahi-Buisson N. (2010). Rett syndrome: Revised diagnostic criteria and nomenclature. *Ann. Neurol.* 68 944–950. 10.1002/ana.22124 21154482 PMC3058521

[B173] Neul J. L. Lane J. B. Lee H. S. Geerts S. Barrish J. O. Annese F. (2014). Developmental delay in Rett syndrome: Data from the natural history study. *J. Neurodev. Disord.* 6:20. 10.1186/1866-1955-6-20 25071871 PMC4112822

[B174] Newpher T. M. Ehlers M. D. (2008). Glutamate receptor dynamics in dendritic microdomains. *Neuron* 58 472–497. 10.1016/j.neuron.2008.04.030 18498731 PMC2572138

[B175] Ni M. Lee A. S. (2007). ER chaperones in mammalian development and human diseases. *FEBS Lett.* 581 3641–3651. 10.1016/j.febslet.2007.04.045 17481612 PMC2040386

[B176] Nikolaienko O. Eriksen M. S. Patil S. Bito H. Bramham C. R. (2017). Stimulus-evoked ERK-dependent phosphorylation of activity-regulated cytoskeleton-associated protein (Arc) regulates its neuronal subcellular localization. *Neuroscience* 360 68–80. 10.1016/j.neuroscience.2017.07.026 28736134

[B177] Nimchinsky E. A. Sabatini B. L. Svoboda K. (2002). Structure and function of dendritic spines. *Annu. Rev. Physiol.* 64 313–353. 10.1146/annurev.physiol.64.081501.160008 11826272

[B178] Nishimura Y. V. Shinoda T. Inaguma Y. Ito H. Nagata K. (2012). Application of in utero electroporation and live imaging in the analyses of neuronal migration during mouse brain development. *Med. Mol. Morphol.* 45 1–6. 10.1007/s00795-011-0557-0 22431177

[B179] Niu S. Renfro A. Quattrocchi C. C. Sheldon M. D’Arcangelo G. (2004). Reelin promotes hippocampal dendrite development through the VLDLR/ApoER2-Dab1 pathway. *Neuron* 41 71–84. 10.1016/s0896-6273(03)00819-5 14715136

[B180] Niu S. Yabut O. D’Arcangelo G. (2008). The reelin signaling pathway promotes dendritic spine development in hippocampal neurons. *J. Neurosci.* 28 10339–10348. 10.1523/JNEUROSCI.1917-08.2008 18842893 PMC2572775

[B181] Nogami T. Beppu H. Tokoro T. Moriguchi S. Shioda N. Fukunaga K. (2011). Reduced expression of the ATRX gene, a chromatin-remodeling factor, causes hippocampal dysfunction in mice. *Hippocampus* 21 678–687. 10.1002/hipo.20782 20865721

[B182] Oldenborg P. A. Zheleznyak A. Fang Y. F. Lagenaur C. F. Gresham H. D. Lindberg F. P. (2000). Role of CD47 as a marker of self on red blood cells. *Science* 288 2051–2054. 10.1126/science.288.5473.2051 10856220

[B183] Olusanya B. O. Wright S. M. Nair M. Boo N. Y. Halpern R. Kuper H. (2020). Global burden of childhood epilepsy, intellectual disability, and sensory impairments. *Pediatrics* 146:e20192623. 10.1542/peds.2019-2623 32554521 PMC7613313

[B184] Omotade O. F. Rui Y. Lei W. Yu K. Hartzell H. C. Fowler V. M. (2018). Tropomodulin Isoform-specific regulation of dendrite development and synapse formation. *J. Neurosci.* 38 10271–10285. 10.1523/JNEUROSCI.3325-17.2018 30301754 PMC6262146

[B185] Osaki Y. Matsuhisa K. Che W. Kaneko M. Asada R. Masaki T. (2019). Calnexin promotes the folding of mutant iduronate 2-sulfatase related to mucopolysaccharidosis type II. *Biochem. Biophys. Res. Commun.* 514 217–223. 10.1016/j.bbrc.2019.04.115 31029429

[B186] Osborn D. Pond H. L. Mazaheri N. Dejardin J. Munn C. J. Mushref K. (2017). Mutations in INPP5K cause a form of congenital muscular dystrophy overlapping marinesco-sjögren syndrome and dystroglycanopathy. *Am. J. Hum. Genet.* 100 537–545. 10.1016/j.ajhg.2017.01.019 28190459 PMC5339112

[B187] Otero J. H. Lizák B. Hendershot L. M. (2010). Life and death of a BiP substrate. *Semin. Cell Dev. Biol.* 21 472–478. 10.1016/j.semcdb.2009.12.008 20026282 PMC2883687

[B188] Pabba M. Wong A. Y. Ahlskog N. Hristova E. Biscaro D. Nassrallah W. (2014). NMDA receptors are upregulated and trafficked to the plasma membrane after sigma-1 receptor activation in the rat hippocampus. *J. Neurosci.* 34 11325–11338. 10.1523/JNEUROSCI.0458-14.2014 25143613 PMC6615506

[B189] Pacheco A. Merianda T. T. Twiss J. L. Gallo G. (2020). Mechanism and role of the intra-axonal calreticulin translation in response to axonal injury. *Exp. Neurol.* 323:113072. 10.1016/j.expneurol.2019.113072 31669485 PMC6909931

[B190] Pan C. L. Howell J. E. Clark S. G. Hilliard M. Cordes S. Bargmann C. I. (2006). Multiple Wnts and frizzled receptors regulate anteriorly directed cell and growth cone migrations in *Caenorhabditis elegans*. *Dev. Cell* 10 367–377. 10.1016/j.devcel.2006.02.010 16516839

[B191] Pan Z. Erkan M. Streit S. Friess H. Kleeff J. (2009). Silencing of GRP94 expression promotes apoptosis in pancreatic cancer cells. *Int. J. Oncol.* 35 823–828. 10.3892/ijo_00000395 19724918

[B192] Parakh S. Atkin J. D. (2015). Novel roles for protein disulphide isomerase in disease states: A double edged sword? *Front. Cell Dev. Biol.* 3:30. 10.3389/fcell.2015.00030 26052512 PMC4439577

[B193] Penke B. Fulop L. Szucs M. Frecska E. (2018). The role of sigma-1 receptor, an intracellular chaperone in neurodegenerative diseases. *Curr. Neuropharmacol.* 16 97–116. 10.2174/1570159X15666170529104323 28554311 PMC5771390

[B194] Perri E. R. Thomas C. J. Parakh S. Spencer D. M. Atkin J. D. (2016). The unfolded protein response and the role of protein disulfide isomerase in neurodegeneration. *Front. Cell Dev. Biol.* 3:80. 10.3389/fcell.2015.00080 26779479 PMC4705227

[B195] Peviani M. Salvaneschi E. Bontempi L. Petese A. Manzo A. Rossi D. (2014). Neuroprotective effects of the sigma-1 receptor (S1R) agonist PRE-084, in a mouse model of motor neuron disease not linked to SOD1 mutation. *Neurobiol. Dis.* 62 218–232. 10.1016/j.nbd.2013.10.010 24141020

[B196] Pfaffenseller B. Wollenhaupt-Aguiar B. Fries G. R. Colpo G. D. Burque R. K. Bristot G. (2014). Impaired endoplasmic reticulum stress response in bipolar disorder: Cellular evidence of illness progression. *Int. J. Neuropsychopharmacol.* 17 1453–1463. 10.1017/S1461145714000443 24800824

[B197] Phillips M. J. Voeltz G. K. (2016). Structure and function of ER membrane contact sites with other organelles. *Nat. Rev. Mol. Cell Biol.* 17 69–82. 10.1038/nrm.2015.8 26627931 PMC5117888

[B198] Pick J. E. Ziff E. B. (2018). Regulation of AMPA receptor trafficking and exit from the endoplasmic reticulum. *Mol. Cell. Neurosci.* 91 3–9. 10.1016/j.mcn.2018.03.004 29545119 PMC6128777

[B199] Pilquil C. Alvandi Z. Opas M. (2020). Calreticulin regulates a switch between osteoblast and chondrocyte lineages derived from murine embryonic stem cells. *J. Biol. Chem.* 295 6861–6875. 10.1074/jbc.RA119.011029 32220932 PMC7242707

[B200] Pobre K. Poet G. J. Hendershot L. M. (2019). The endoplasmic reticulum (ER) chaperone BiP is a master regulator of ER functions: Getting by with a little help from ERdj friends. *J. Biol. Chem.* 294 2098–2108. 10.1074/jbc.REV118.002804 30563838 PMC6369273

[B201] Polanczyk G. V. Willcutt E. G. Salum G. A. Kieling C. Rohde L. A. (2014). ADHD prevalence estimates across three decades: An updated systematic review and meta-regression analysis. *Int. J. Epidemiol.* 43 434–442. 10.1093/ije/dyt261 24464188 PMC4817588

[B202] Polanczyk G. de Lima M. S. Horta B. L. Biederman J. Rohde L. A. (2007). The worldwide prevalence of ADHD: A systematic review and metaregression analysis. *Am. J. Psychiatry* 164 942–948. 10.1176/ajp.2007.164.6.942 17541055

[B203] Prigge C. L. Kay J. N. (2018). Dendrite morphogenesis from birth to adulthood. *Curr. Opin. Neurobiol.* 53 139–145. 10.1016/j.conb.2018.07.007 30092409 PMC6242770

[B204] Rapoport T. A. (2007). Protein translocation across the eukaryotic endoplasmic reticulum and bacterial plasma membranes. *Nature* 450 663–669. 10.1038/nature06384 18046402

[B205] Rauch F. Prud’homme J. Arabian A. Dedhar S. St-Arnaud R. (2000). Heart, brain, and body wall defects in mice lacking calreticulin. *Exp. Cell Res.* 256 105–111. 10.1006/excr.2000.4818 10739657

[B206] Reyes S. T. Deacon R. Guo S. G. Altimiras F. J. Castillo J. B. van der Wildt B. (2021). Effects of the sigma-1 receptor agonist blarcamesine in a murine model of fragile X syndrome: Neurobehavioral phenotypes and receptor occupancy. *Sci. Rep.* 11:17150. 10.1038/s41598-021-94079-7 34433831 PMC8387417

[B207] Riquelme P. A. Drapeau E. Doetsch F. (2008). Brain micro-ecologies: Neural stem cell niches in the adult mammalian brain. *Philos. Trans. R. Soc. Lond. B Biol. Sci.* 363 123–137. 10.1098/rstb.2006.2016 17322003 PMC2605490

[B208] Rizzuto R. Duchen M. R. Pozzan T. (2004). Flirting in little space: The ER/mitochondria Ca2+ liaison. *Sci. STKE* 2004:re1. 10.1126/stke.2152004re1 14722345

[B209] Robson M. J. Turner R. C. Naser Z. J. McCurdy C. R. O’Callaghan J. P. Huber J. D. (2014). SN79, a sigma receptor antagonist, attenuates methamphetamine-induced astrogliosis through a blockade of OSMR/gp130 signaling and STAT3 phosphorylation. *Exp. Neurol.* 254 180–189. 10.1016/j.expneurol.2014.01.020 24508558 PMC4241368

[B210] Roderick H. L. Lechleiter J. D. Camacho P. (2000). Cytosolic phosphorylation of calnexin controls intracellular Ca(2+) oscillations via an interaction with SERCA2b. *J. Cell Biol.* 149 1235–1248. 10.1083/jcb.149.6.1235 10851021 PMC2175122

[B211] Roos A. Buchkremer S. Kollipara L. Labisch T. Gatz C. Zitzelsberger M. (2014). Myopathy in Marinesco-Sjögren syndrome links endoplasmic reticulum chaperone dysfunction to nuclear envelope pathology. *Acta Neuropathol.* 127 761–777. 10.1007/s00401-013-1224-4 24362440

[B212] Ropers H. H. (2010). Genetics of early onset cognitive impairment. *Annu. Rev. Genomics Hum. Genet.* 11 161–187. 10.1146/annurev-genom-082509-141640 20822471

[B213] Rossi S. De Chiara V. Musella A. Cozzolino M. Bernardi G. Maccarrone M. (2010). Abnormal sensitivity of cannabinoid CB1 receptors in the striatum of mice with experimental amyotrophic lateral sclerosis. *Amyotroph. Lateral* 11 83–90. 10.3109/17482960902977954 19452308

[B214] Rosso I. M. Killgore W. D. Cintron C. M. Gruber S. A. Tohen M. Yurgelun-Todd D. A. (2007). Reduced amygdala volumes in first-episode bipolar disorder and correlation with cerebral white matter. *Biol. Psychiatry* 61 743–749. 10.1016/j.biopsych.2006.07.035 17123471

[B215] Rubio M. E. Wenthold R. J. (1999). Calnexin and the immunoglobulin binding protein (BiP) coimmunoprecipitate with AMPA receptors. *J. Neurochem.* 73 942–948. 10.1046/j.1471-4159.1999.0730942.x 10461883

[B216] Ruegsegger C. Saxena S. (2016). Proteostasis impairment in ALS. *Brain Res.* 1648(Pt B) 571–579. 10.1016/j.brainres.2016.03.032 27033833

[B217] Ruscher K. Inácio A. R. Valind K. Rowshan Ravan A. Kuric E. Wieloch T. (2012). Effects of the sigma-1 receptor agonist 1-(3,4-dimethoxyphenethyl)-4-(3-phenylpropyl)-piperazine dihydro-chloride on inflammation after stroke. *PLoS One* 7:e45118. 10.1371/journal.pone.0045118 23028794 PMC3445585

[B218] Ruscher K. Shamloo M. Rickhag M. Ladunga I. Soriano L. Gisselsson L. (2011). The sigma-1 receptor enhances brain plasticity and functional recovery after experimental stroke. *Brain* 134(Pt 3) 732–746. 10.1093/brain/awq367 21278085

[B219] Rutter M. Kim-Cohen J. Maughan B. (2006). Continuities and discontinuities in psychopathology between childhood and adult life. *J. Child Psychol. Psychiatry* 47 276–295. 10.1111/j.1469-7610.2006.01614.x 16492260

[B220] Ryskamp D. A. Korban S. Zhemkov V. Kraskovskaya N. Bezprozvanny I. (2019). Neuronal sigma-1 receptors: Signaling functions and protective roles in neurodegenerative diseases. *Front. Neurosci.* 13:862. 10.3389/fnins.2019.00862 31551669 PMC6736580

[B221] Sacco R. Gabriele S. Persico A. M. (2015). Head circumference and brain size in autism spectrum disorder: A systematic review and meta-analysis. *Psychiatry Res.* 234 239–251. 10.1016/j.pscychresns.2015.08.016 26456415

[B222] Salinas P. C. Zou Y. (2008). Wnt signaling in neural circuit assembly. *Annu. Rev. Neurosci.* 31 339–358. 10.1146/annurev.neuro.31.060407.125649 18558859

[B223] Samaco R. C. McGraw C. M. Ward C. S. Sun Y. Neul J. L. Zoghbi H. Y. (2013). Female Mecp2(+/-) mice display robust behavioral deficits on two different genetic backgrounds providing a framework for pre-clinical studies. *Hum. Mol. Genet.* 22 96–109. 10.1093/hmg/dds406 23026749 PMC3522402

[B224] Schmidt H. R. Kruse A. C. (2019). The molecular function of σ receptors: Past, present, and future. *Trends Pharmacol. Sci.* 40 636–654. 10.1016/j.tips.2019.07.006 31387763 PMC6748033

[B225] Schwarz D. S. Blower M. D. (2016). The endoplasmic reticulum: Structure, function and response to cellular signaling. *Cell. Mol. Life Sci.* 73 79–94. 10.1007/s00018-015-2052-6 26433683 PMC4700099

[B226] Shepherd J. D. Bear M. F. (2011). New views of Arc, a master regulator of synaptic plasticity. *Nat. Neurosci.* 14 279–284. 10.1038/nn.2708 21278731 PMC8040377

[B227] Shih Y. Y. Nakagawara A. Lee H. Juan H. F. Jeng Y. M. Lin D. T. (2012). Calreticulin mediates nerve growth factor-induced neuronal differentiation. *J. Mol. Neurosci.* 47 571–581. 10.1007/s12031-011-9683-3 22147490

[B228] Shioda N. Beppu H. Fukuda T. Li E. Kitajima I. Fukunaga K. (2011). Aberrant calcium/calmodulin-dependent protein kinase II (CaMKII) activity is associated with abnormal dendritic spine morphology in the ATRX mutant mouse brain. *J. Neurosci.* 31 346–358. 10.1523/JNEUROSCI.4816-10.2011 21209221 PMC6622766

[B229] Siegert R. Leroux M. R. Scheufler C. Hartl F. U. Moarefi I. (2000). Structure of the molecular chaperone prefoldin: Unique interaction of multiple coiled coil tentacles with unfolded proteins. *Cell* 103 621–632. 10.1016/s0092-8674(00)00165-3 11106732

[B230] Smith S. M. Renden R. von Gersdorff H. (2008). Synaptic vesicle endocytosis: Fast and slow modes of membrane retrieval. *Trends Neurosci.* 31 559–568. 10.1016/j.tins.2008.08.005 18817990 PMC3626563

[B231] So J. Warsh J. J. Li P. P. (2007). Impaired endoplasmic reticulum stress response in B-lymphoblasts from patients with bipolar-I disorder. *Biol. Psychiatry* 62 141–147. 10.1016/j.biopsych.2006.10.014 17217928

[B232] Sokolova E. Oerlemans A. M. Rommelse N. N. Groot P. Hartman C. A. Glennon J. C. (2017). A causal and mediation analysis of the comorbidity between attention deficit hyperactivity disorder (ADHD) and autism spectrum disorder (ASD). *J. Autism Dev. Disord.* 47 1595–1604. 10.1007/s10803-017-3083-7 28255761 PMC5432632

[B233] Solheim J. C. Harris M. R. Kindle C. S. Hansen T. H. (1997). Prominence of beta 2-microglobulin, class I heavy chain conformation, and tapasin in the interactions of class I heavy chain with calreticulin and the transporter associated with antigen processing. *J. Immunol.* 158 2236–2241. 9036970

[B234] Stapleton M. Arunkumar N. Kubaski F. Mason R. W. Tadao O. Tomatsu S. (2018). Clinical presentation and diagnosis of mucopolysaccharidoses. *Mol. Genet. Metab.* 125 4–17. 10.1016/j.ymgme.2018.01.003 30057281

[B235] Stranahan A. M. Erion J. R. Wosiski-Kuhn M. (2013). Reelin signaling in development, maintenance, and plasticity of neural networks. *Ageing Res. Rev.* 12 815–822. 10.1016/j.arr.2013.01.005 23352928 PMC4475282

[B236] Su T. P. Hayashi T. Maurice T. Buch S. Ruoho A. E. (2010). The sigma-1 receptor chaperone as an inter-organelle signaling modulator. *Trends Pharmacol. Sci.* 31 557–566. 10.1016/j.tips.2010.08.007 20869780 PMC2993063

[B237] Su T. P. Su T. C. Nakamura Y. Tsai S. Y. (2016). The sigma-1 receptor as a pluripotent modulator in living systems. *Trends Pharmacol. Sci.* 37 262–278. 10.1016/j.tips.2016.01.003 26869505 PMC4811735

[B238] Sudhof T. C. (2004). The synaptic vesicle cycle. *Annu. Rev. Neurosci.* 27 509–547. 10.1146/annurev.neuro.26.041002.131412 15217342

[B239] Szabadkai G. Bianchi K. Várnai P. De Stefani D. Wieckowski M. R. Cavagna D. (2006). Chaperone-mediated coupling of endoplasmic reticulum and mitochondrial Ca2+ channels. *J. Cell Biol.* 175 901–911. 10.1083/jcb.200608073 17178908 PMC2064700

[B240] Tabata H. Nakajima K. (2001). Efficient in utero gene transfer system to the developing mouse brain using electroporation: Visualization of neuronal migration in the developing cortex. *Neuroscience* 103 865–872. 10.1016/s0306-4522(01)00016-1 11301197

[B241] Tahmaz I. Shahmoradi Ghahe S. Topf U. (2022). Prefoldin function in cellular protein homeostasis and human diseases. *Front. Cell Dev. Biol.* 9:816214. 10.3389/fcell.2021.816214 35111762 PMC8801880

[B242] Takeuchi T. (2018). Non-cell autonomous maintenance of proteostasis by molecular chaperones and its molecular mechanism. *Biol. Pharm. Bull.* 41 843–849. 10.1248/bpb.b18-00141 29863073

[B243] Tan Q. Zoghbi H. Y. (2019). Mouse models as a tool for discovering new neurological diseases. *Neurobiol. Learn. Mem.* 165:106902. 10.1016/j.nlm.2018.07.006 30030131

[B244] Tashiro E. Zako T. Muto H. Itoo Y. Sörgjerd K. Terada N. (2013). Prefoldin protects neuronal cells from polyglutamine toxicity by preventing aggregation formation. *J. Biol. Chem.* 288 19958–19972. 10.1074/jbc.M113.477984 23720755 PMC3707696

[B245] Thapar A. Cooper M. Rutter M. (2017). Neurodevelopmental disorders. *Lancet Psychiatry* 4 339–346. 10.1016/S2215-0366(16)30376-5 27979720

[B246] Tjoelker L. W. Seyfried C. E. Eddy R. L. Jr. Byers M. G. Shows T. B. Calderon J. (1994). Human, mouse, and rat calnexin cDNA cloning: Identification of potential calcium binding motifs and gene localization to human chromosome 5. *Biochemistry* 33 3229–3236. 10.1021/bi00177a013 8136357

[B247] Torpe N. Gopal S. Baltaci O. Rella L. Handley A. Korswagen H. C. (2019). A protein disulfide isomerase controls neuronal migration through regulation of Wnt secretion. *Cell Rep.* 26 3183–3190.e5. 10.1016/j.celrep.2019.02.072 30893592

[B248] Tripp G. Wickens J. R. (2009). Neurobiology of ADHD. *Neuropharmacology* 57 579–589. 10.1016/j.neuropharm.2009.07.026 19627998

[B249] Trujillo C. M. Alonso A. Delgado A. C. Damas C. (2005). The rostral and caudal boundaries of the diencephalon. *Brain Res. Brain Res. Rev.* 49 202–210. 10.1016/j.brainresrev.2005.01.002 16111550

[B250] Tsai S. Y. Hayashi T. Harvey B. K. Wang Y. Wu W. W. Shen R. F. (2009). Sigma-1 receptors regulate hippocampal dendritic spine formation via a free radical-sensitive mechanism involving Rac1xGTP pathway. *Proc. Natl. Acad. Sci. U.S.A.* 106 22468–22473. 10.1073/pnas.0909089106 20018732 PMC2792161

[B251] Tsuboyama K. Tadakuma H. Tomari Y. (2018). Conformational activation of argonaute by distinct yet coordinated actions of the Hsp70 and Hsp90 chaperone systems. *Mol. Cell* 70 722–729.e4. 10.1016/j.molcel.2018.04.010 29775584

[B252] Turano C. Coppari S. Altieri F. Ferraro A. (2002). Proteins of the PDI family: Unpredicted non-ER locations and functions. *J. Cell. Physiol.* 193 154–163. 10.1002/jcp.10172 12384992

[B253] Turrero García M. Harwell C. C. (2017). Radial glia in the ventral telencephalon. *FEBS Lett.* 591 3942–3959. 10.1002/1873-3468.12829 28862741 PMC5747302

[B254] Turrigiano G. G. (2008). The self-tuning neuron: Synaptic scaling of excitatory synapses. *Cell* 135 422–435. 10.1016/j.cell.2008.10.008 18984155 PMC2834419

[B255] Tyson J. R. Stirling C. J. (2000). LHS1 and SIL1 provide a lumenal function that is essential for protein translocation into the endoplasmic reticulum. *EMBO J.* 19 6440–6452. 10.1093/emboj/19.23.6440 11101517 PMC305876

[B256] van Schadewijk A. van’t Wout E. F. Stolk J. Hiemstra P. S. (2012). A quantitative method for detection of spliced X-box binding protein-1 (XBP1) mRNA as a measure of endoplasmic reticulum (ER) stress. *Cell Stress Chaperones* 17 275–279. 10.1007/s12192-011-0306-2 22038282 PMC3273559

[B257] Villard V. Espallergues J. Keller E. Vamvakides A. Maurice T. (2011). Anti-amnesic and neuroprotective potentials of the mixed muscarinic receptor/sigma 1 (σ1) ligand ANAVEX2-73, a novel aminotetrahydrofuran derivative. *J. Psychopharmacol.* 25 1101–1117. 10.1177/0269881110379286 20829307

[B258] Vuppalanchi D. Coleman J. Yoo S. Merianda T. T. Yadhati A. G. Hossain J. (2010). Conserved 3’-untranslated region sequences direct subcellular localization of chaperone protein mRNAs in neurons. *J. Biol. Chem.* 285 18025–18038. 10.1074/jbc.M109.061333 20308067 PMC2878564

[B259] Wang J. Lee J. Liem D. Ping P. (2017). HSPA5 gene encoding Hsp70 chaperone BiP in the endoplasmic reticulum. *Gene* 618 14–23. 10.1016/j.gene.2017.03.005 28286085 PMC5632570

[B260] Wang M. Wey S. Zhang Y. Ye R. Lee A. S. (2009). Role of the unfolded protein response regulator GRP78/BiP in development, cancer, and neurological disorders. *Antioxid. Redox Signal.* 11 2307–2316. 10.1089/ars.2009.2485 19309259 PMC2819800

[B261] Wang T. Liu K. Li Z. Xu Y. Liu Y. Shi W. (2017). Prevalence of attention deficit/hyperactivity disorder among children and adolescents in China: A systematic review and meta-analysis. *BMC Psychiatry* 17:32. 10.1186/s12888-016-1187-9 28103833 PMC5244567

[B262] Wang Z. H. Chen Y. X. Zhang C. M. Wu L. Yu Z. Cai X. L. (2011). Intermittent hypobaric hypoxia improves postischemic recovery of myocardial contractile function via redox signaling during early reperfusion. *Am. J. Physiol. Heart Circ. Physiol.* 301 H1695–H1705. 10.1152/ajpheart.00276.2011 21821784

[B263] Wang Z. Hong Y. Zou L. Zhong R. Zhu B. Shen N. (2014). Reelin gene variants and risk of autism spectrum disorders: An integrated meta-analysis. *Am. J. Med. Genet. B Neuropsychiatr. Genet.* 165B 192–200. 10.1002/ajmg.b.32222 24453138

[B264] Weiss L. A. Shen Y. Korn J. M. Arking D. E. Miller D. T. Fossdal R. (2008). Association between microdeletion and microduplication at 16p11.2 and autism. *N. Engl. J. Med.* 358 667–675. 10.1056/NEJMoa075974 18184952

[B265] Whangbo J. Kenyon C. (1999). A Wnt signaling system that specifies two patterns of cell migration in *C. elegans*. *Mol. Cell* 4 851–858. 10.1016/s1097-2765(00)80394-9 10619031

[B266] Wilkinson B. Gilbert H. F. (2004). Protein disulfide isomerase. *Biochim. Biophys. Acta* 1699 35–44. 10.1016/j.bbapap.2004.02.017 15158710

[B267] Willert K. Nusse R. (2012). Wnt proteins. *Cold Spring Harb. Perspect. Biol.* 4:a007864. 10.1101/cshperspect.a007864 22952392 PMC3428774

[B268] Willis D. Li K. W. Zheng J. Q. Chang J. H. Smit A. B. Kelly T. (2005). Differential transport and local translation of cytoskeletal, injury-response, and neurodegeneration protein mRNAs in axons. *J. Neurosci.* 25 778–791. 10.1523/JNEUROSCI.4235-04.2005 15673657 PMC6725618

[B269] Wilson P. J. Morris C. P. Anson D. S. Occhiodoro T. Bielicki J. Clements P. R. (1990). Hunter syndrome: Isolation of an iduronate-2-sulfatase cDNA clone and analysis of patient DNA. *Proc. Natl. Acad. Sci. U.S.A.* 87 8531–8535. 10.1073/pnas.87.21.8531 2122463 PMC54990

[B270] Wraith J. E. Rogers J. G. Danks D. M. (1987). The mucopolysaccharidoses. *Aust. Paediatr. J.* 23 329–334. 10.1111/j.1440-1754.1987.tb00284.x 3124802

[B271] Xavier J. M. Rodrigues C. M. Solá S. (2016). Mitochondria: Major regulators of neural development. *Neuroscientist* 22 346–358. 10.1177/1073858415585472 25948649

[B272] Yamaguchi K. Shioda N. Yabuki Y. Zhang C. Han F. Fukunaga K. (2018). SA4503, a potent sigma-1 receptor ligand, ameliorates synaptic abnormalities and cognitive dysfunction in a mouse model of ATR-X syndrome. *Int. J. Mol. Sci.* 19:2811. 10.3390/ijms19092811 30231518 PMC6163584

[B273] Yan M. Li J. Sha B. (2011). Structural analysis of the sil1-bip complex reveals the mechanism for sil1 to function as a nucleotide-exchange factor. *Biochem. J.* 438 447–455. 10.1042/BJ20110500 21675960

[B274] Yashiro K. Philpot B. D. (2008). Regulation of NMDA receptor subunit expression and its implications for LTD, LTP, and metaplasticity. *Neuropharmacology* 55 1081–1094. 10.1016/j.neuropharm.2008.07.046 18755202 PMC2590778

[B275] Yoshikawa S. McKinnon R. D. Kokel M. Thomas J. B. (2003). Wnt-mediated axon guidance via the *Drosophila* derailed receptor. *Nature* 422 583–588. 10.1038/nature01522 12660735

[B276] Zablotsky B. Black L. I. Maenner M. J. Schieve L. A. Blumberg S. J. (2015). Estimated prevalence of autism and other developmental disabilities following questionnaire changes in the 2014 national health interview survey. *Natl. Health Stat. Rep.* 87 1–20. 26632847

[B277] Zarrei M. Burton C. L. Engchuan W. Young E. J. Higginbotham E. J. MacDonald J. R. (2019). A large data resource of genomic copy number variation across neurodevelopmental disorders. *NPJ Genom. Med.* 4:26. 10.1038/s41525-019-0098-3 31602316 PMC6779875

[B278] Zeeshan H. M. Lee G. H. Kim H. R. Chae H. J. (2016). Endoplasmic reticulum stress and associated ROS. *Int. J. Mol. Sci.* 17:327. 10.3390/ijms17030327 26950115 PMC4813189

[B279] Zhang X. Abreu J. G. Yokota C. MacDonald B. T. Singh S. Coburn K. L. (2012). Tiki1 is required for head formation via Wnt cleavage-oxidation and inactivation. *Cell* 149 1565–1577. 10.1016/j.cell.2012.04.039 22726442 PMC3383638

[B280] Zhang C. L. Feng Z. J. Liu Y. Ji X. H. Peng J. Y. Zhang X. H. (2012). Methylphenidate enhances NMDA-receptor response in medial prefrontal cortex via sigma-1 receptor: A novel mechanism for methylphenidate action. *PLoS One* 7:e51910. 10.1371/journal.pone.0051910 23284812 PMC3527396

[B281] Zhang K. Kaufman R. J. (2008). From endoplasmic-reticulum stress to the inflammatory response. *Nature* 454 455–462. 10.1038/nature07203 18650916 PMC2727659

[B282] Zhang X. M. Yan X. Y. Zhang B. Yang Q. Ye M. Cao W. (2015). Activity-induced synaptic delivery of the GluN2A-containing NMDA receptor is dependent on endoplasmic reticulum chaperone bip and involved in fear memory. *Cell Res.* 25 818–836. 10.1038/cr.2015.75 26088419 PMC4493282

[B283] Zhao P. Ignacio S. Beattie E. C. Abood M. E. (2008). Altered presymptomatic AMPA and cannabinoid receptor trafficking in motor neurons of ALS model mice: Implications for excitotoxicity. *Eur. J. Neurosci.* 27 572–579. 10.1111/j.1460-9568.2008.06041.x 18279310 PMC3991137

[B284] Zhemkov V. Geva M. Hayden M. R. Bezprozvanny I. (2021). Sigma-1 receptor (S1R) interaction with cholesterol: Mechanisms of S1R activation and its role in neurodegenerative diseases. *Int. J. Mol. Sci.* 22:4082. 10.3390/ijms22084082 33920913 PMC8071319

[B285] Zheng P. (2009). Neuroactive steroid regulation of neurotransmitter release in the CNS: Action, mechanism and possible significance. *Prog. Neurobiol.* 89 134–152. 10.1016/j.pneurobio.2009.07.001 19595736

